# Ethical issues in public health surveillance: a systematic qualitative review

**DOI:** 10.1186/s12889-017-4200-4

**Published:** 2017-04-04

**Authors:** Corinna Klingler, Diego Steven Silva, Christopher Schuermann, Andreas Alois Reis, Abha Saxena, Daniel Strech

**Affiliations:** 1 Institute of Ethics, History and Theory of Medicine at LMU Munich, Lessingstr. 2, 80336 Munich, Germany; 2 Faculty of Health Sciences, Simon Fraser University, Blusson Hall, Room 11008, 8888 University Drive, Burnaby, B.C V5A 1S6 Canada; 3 Institute for History, Ethics and Philosophy of Medicine, Hannover Medical School, Carl-Neuberg-Str. 1, 30625 Hannover, Germany; 4 Global Health Ethics Unit, World Health Organization, Avenue Appia 20, Geneva, GE 1211 Switzerland

**Keywords:** Public health surveillance, Ethics, Informed consent, Systematic review, Qualitative evidence synthesis

## Abstract

**Background:**

Public health surveillance is not ethically neutral and yet, ethics guidance and training for surveillance programmes is sparse. Development of ethics guidance should be based on comprehensive and transparently derived overviews of ethical issues and arguments. However, existing overviews on surveillance ethics are limited in scope and in how transparently they derived their results. Our objective was accordingly to provide an overview of ethical issues in public health surveillance; in addition, to list the arguments put forward with regards to arguably the most contested issue in surveillance, that is whether to obtain informed consent.

**Methods:**

Ethical issues were defined based on principlism. We assumed an ethical issue to arise in surveillance when a relevant normative principle is not adequately considered or two principles come into conflict. We searched Pubmed and Google Books for relevant publications. We analysed and synthesized the data using qualitative content analysis.

**Results:**

Our search strategy retrieved 525 references of which 83 were included in the analysis. We identified 86 distinct ethical issues arising in the different phases of the surveillance life-cycle. We further identified 20 distinct conditions that make it more or less justifiable to forego informed consent procedures.

**Conclusions:**

This is the first systematic qualitative review of ethical issues in public health surveillance resulting in a comprehensive ethics matrix that can inform guidelines, reports, strategy papers, and educational material and raise awareness among practitioners.

**Electronic supplementary material:**

The online version of this article (doi:10.1186/s12889-017-4200-4) contains supplementary material, which is available to authorized users.

## Background

Surveillance is often referred to as the foundation or eyes of public health [1]. The World Health Organization (WHO) defines public health surveillance as “systematic ongoing collection, collation and analysis of data for public health purposes and the timely dissemination of public health information for assessment and public health response as necessary” [2]. Although other organizations define surveillance slightly differently, the goal of informing public health practice is an essential element of most definitions. Public health surveillance activities can be differentiated along various dimensions [3]. The types of diseases surveilled include infectious diseases as well as non-infectious conditions like chronic diseases or risks of negative environmental exposure to health. It can rely on various data sources like reporting by health professionals, health surveys or social media data, and it informs various types of activities from education to the use of restrictive measures.

It is important to note at the outset that public health surveillance involves considerable ethical challenges. In the normative and empirical literature various ethical considerations in surveillance have been highlighted. The fact that often surveillance data is collected without informed consent of those affected has been addressed frequently, provoking debates about whether this constitutes an unjustified infringement of privacy or autonomy rights although it allows the production of a more complete and reliable data set [3–5]. Especially in the context of HIV/AIDS, experts have warned that data collection strategies targeting certain vulnerable groups or public release of data identifying high-risk groups might lead to stigmatization and discrimination [6–8].

Although surveillance raises several ethical issues, few government or institutional policy documents exist that provide real-world guidance regarding how to address these ethical challenges. Most of the normative policy documents produced so far focus on specific disease areas of surveillance like HIV/AIDS or occupational health [7, 9, 10] or discuss public health projects in generalities [11, 12]. Given this existing paucity, and growing demands from different surveillance programmes, the WHO responded by initiating a process for developing a comprehensive ethics guidance on public health surveillance.

While scientific policy and guideline development has been professionalized in the last decade via standards e.g. for grading recommendations [13] or managing conflicts of interests [14], the practice of ethics guideline development has so far not been subject to the same procedural and substantive standards for ensuring high quality recommendations [15–17]. A core challenge for the guideline developers is identifying relevant issues and arguments [18, 19]. Ideally, guidelines should be based on a transparent and comprehensive assessment of ethical issues that may arise in the chosen context to allow a rational and fair selection of issues to be addressed [20]. Furthermore, a full overview of arguments put forward for how to handle certain issues would be essential to ensure no important (argumentative) strategies are missed. Systematic (qualitative) literature reviews enable structured and methodologically informed identification and synthesis of the relevant literature with regards to pre-specified questions of interest and can thereby assist in articulating possible issues and arguments that ought to be considered and used (or explicitly ignored) when developing ethics guidance.

So far no systematic qualitative reviews of ethical issues in public health surveillance have been conducted. There are a few introductory book chapters or overview articles published identifying key ethical issues in surveillance [3, 21–23]. Furthermore, some papers are devoted exclusively to discussing the very prominent topic of informed consent [24, 25] with one publication specifying certain conditions that – if fulfilled – could justify foregoing informed consent [5]. However, all those articles are narrative in type, did not employ a methodology to ensure comprehensiveness, did not explain how they selected certain issues or conditions discussed, and were generally limited in scope due to their focus on a few particular issues or arguments.^1^ A systematic qualitative review of ethical issues in surveillance could help fill existing gaps.

This systematic qualitative review was undertaken to inform the process of developing WHO guidelines on ethical issues in public health surveillance. Accordingly, the purpose of this systematic qualitative review was to give a comprehensive overview of ethical issues in public health surveillance as discussed in the academic literature. Furthermore, an additional goal was to provide a comprehensive list of arguments raised for resolving the seemingly most prominent issue in surveillance debates, namely informed consent. The purpose of this review is purely descriptive; it does not evaluate and weigh the arguments raised. We abstained from further analysis as there are no universally accepted criteria or procedures for evaluating and synthesizing arguments as there are for e.g. quantitative data (like GRADE [13]). The review can only be a starting point and decision-makers will have to implement further evaluative steps taking into consideration the relevant contextual factors. This review not only informed WHO’s guidelines, but can also support reports, strategy papers, and educational material on public health surveillance. It will hopefully also raise awareness among practitioners with regards to the variety of ethical issues they need to consider when planning, implementing, and executing public health surveillance systems.

## Methods

The description of methods follows the Preferred Reporting Items for Systematic Reviews and Meta-Analyses (short: PRISMA) Statement as far as applicable to qualitative evidence syntheses [26] (see Additional file 1 for completed PRISMA checklist). No review protocol was published beforehand.

### Inclusion criteria

For this review, we had to define two contentious terms based on which we could formulate clear inclusion criteria – (a) ethical issues and (b) public health surveillance. Our definition of ethical issues is based on principlism [27] which is commonly employed in bioethics and has been used successfully in other systematic qualitative ethics reviews [18, 19, 28]. Most public health ethics frameworks are developed in this tradition and accordingly identify principles that are understood as prima facie binding and action-guiding [29]. One such framework – chosen by us because it was built on the experience with and integrated the principles elaborated in previously published frameworks – identifies five guiding principles: beneficence, non-maleficence, respect for autonomy, equity and efficiency [30]. With respect to those principles we assume that an ethical issue arises when (a) at least one of those principles is not adequately considered (e.g. communities experience stigmatization by dissemination of sensible surveillance data which would constitute a harm), which we referred to as **risks** 2 or (b) two or more of these principles are in **conflict** (e.g. the informed consent issue: surveillance activity can only realize public health benefit when violating the confidentiality of medical data and thereby patient autonomy).

In contrast to other ethics reviews, we were furthermore interested in strategies for resolving issues and therefore also extracted and categorized safeguards (defined as strategies to prevent risks from materializing) and conditions (meant as arguments raised to give one of two conflicting principles precedence over the other); however, those findings are not fully presented here as the information generated was excessive for the format of a journal publication. We will only describe our findings with regards to informed consent since it is the seemingly most contested issue.

For public health surveillance, we adopted the definition given in the introduction as formulated in the International Health Regulations of the World Health Organization [2] – an international point of reference. Due to the composition of our research team, we only included publications in English, Spanish or German. Furthermore, publications needed to be a journal article, book or book chapter, or a report from a governmental institution to facilitate a rigorous and reproducible search strategy.

### Search strategy and data sources

Our search strategy was developed in cooperation with experts of the WHO Guideline Development Group for Ethical Issues in Public Health Surveillance. The search was comprised of the term “ethics” and its synonyms, terms for specific ethical issues that experts identified as relevant in this context, as well as the term “public health surveillance”, its synonyms and specific types of surveillance. The search strategy for PubMed is presented in Table 1 as required by PRISMA. The search was conducted in February 2015. Additionally, we searched Google Books [31] with a search string combining ethics, public health surveillance and their synonyms. This strategy produced more than 20,000 hits. Due to the large number and because Google Books sorts hits by relevance, we only included the first 100 publications. The Google Book’s sorting algorithm had face validity as we found among the first 20 hits all important publication of which we were already aware; upon an initial review, past the first 20 hits it seemed that almost none of the publications applied to our search. The Google Books search was conducted in March 2015.

**Table 1:** Search strategy in PubMed

Ethics	1	ethics [Mesh Terms] OR morals [Mesh Terms] OR ethic* [Title/Abstract]
	2	human rights [Mesh Terms] OR “human rights” [Title/Abstract]
	3	government regulation [Mesh Terms] OR regulation [Title/Abstract] OR governance [Title/Abstract]
	4	1 OR 2 OR 3
Specific ethical issues	5	informed consent [Mesh Terms] OR consent [Title/Abstract]
	6	“no treatment” [Title/Abstract] OR untreatable [Title/Abstract] OR incurable [Title/Abstract]
	7	“follow-up care” [Title/Abstract] OR “level of care” [Title/Abstract] or “standard of care” [Title/Abstract]
	8	(4 OR 5 OR 6 OR 7)
Public health surveillance	9	public health surveillance [Mesh Terms] OR “public health surveillance” [Title/Abstract]
	10	biosurveillance [Mesh Terms] OR health information systems [Mesh Terms] OR biosurveillance [Title/Abstract] OR “disease surveillance” [Title/Abstract] OR “health information system” [Title/Abstract] OR “epidemiological surveillance” [Title/Abstract]
	11	sentinel surveillance [Mesh Terms] OR “case-based surveillance” [Title/Abstract] OR “event-based surveillance” [Title/Abstract] OR “syndromic surveillance” [Title/Abstract] OR “sentinel surveillance” [Title/Abstract] OR “epidemic intelligence” [Title/Abstract]
	12	(9 OR 10 OR 11)
	13	8 AND 12

**Table 2:** Ethical issues in public health surveillance

Stage in the process	THEMES (highest abstraction level)	
	Code	Subcode (lowest abstraction level)
Background issues	ISSUES RELATED TO CHOICE OF FRAMEWORK FOR CONDUCTING PUBLIC HEALTH SURVEILLANCE	
	Risk of misguided judgement due to lacking ethical framework	Lacking ethical framework for using online data sources
		Lacking ethical framework for how to treat data of the deceased
	Risk of misguided judgement due to using inappropriate ethical framework	Using research ethics framework (because criteria for differentiating research and surveillance are missing)
		Employing the research vs. practice paradigm that lacks moral salience
		Using clinical ethics framework
		Using health security framework
	Issues related to scientific standards for evidence generation	Conflict between different knowledge systems
		Risk of choosing framework for evidence generation that hinders production and use of relevant data
	RISK OF NOT FULFILLING PRECONDITONS FOR SUCCESSFUL PUBLIC HEALTH SURVEILLANCE	
	Risk of barriers hindering development of technology to improve effectiveness and efficiency of surveillance	Lacking funding for technology development
		Lacking necessary multidisciplinary collaboration for technology development
	Risk of not producing sufficiently robust evidence on effective surveillance methods	
Issues in system design and implementation	ISSUES OF DECIDING WHICH PUBLIC HEALTH SURVEILLANC SYSTEM SHOULD BE REALIZED	
	Conflicts of priority setting between different public health programs	Prioritizing between different public health surveillance systems
		Prioritizing between surveillance activity and other public health activities
		Prioritizing potential emerging threats or sustained health issues
	Risk of wasting resources by prioritizing surveillance systems	Prioritizing disease areas important for developed nations instead of areas of high need
		Prioritizing surveillance systems where other investments would serve public health better
	ISSUES OF ADEQUATELY DESIGNING A PUBLIC HEALTH SURVEILLANCE SYSTEM	
	Conflicts of priority setting within the design of a surveillance program	Prioritizing comprehensiveness and accuracy of data or efficiency of surveillance system
		Prioritizing efficiency by minimizing costs or security of data protection when employing digital technology
		Prioritizing early detection of events or efficiency trough reduction of false-positive alarms
		Prioritizing maximizing amount and utility of data or security of private information by limiting data collected
		Prioritizing harmonization of methods to improve sharing arrangements or tailoring to specific purpose
	Risk of making poor choices in design of the surveillance system	Not adequately considering equity issues in surveillance system
		Not adequately tailored to the purpose and context of surveillance
		Not employing health information technology and other promising tools for improvement of surveillance activity
		Not adequately coordinating and integrating surveillance initiatives with other services – especially in developing countries
		Not involving communities in development and implementation of surveillance systems
		Commissioning actors that work ineffectively, inefficiently or unethically with running of surveillance system
		Setting up surveillance systems that are inherently unsustainable, unreliable or insensitive (without adequate safeguards in place)
	RISKS INVOLVED IN IMPLEMENTING AND RUNNING A PUBLIC HEALTH SURVEILLANCE SYSTEM	
	Risk of inadequate legal regulation and governance structures for surveillance project	Inconsistent or overly complex legal guidance complicating effective and ethical implementation – especially for projects implemented across jurisdictions
		No ethical review mechanism ensuring ethical obligations are followed – especially for projects involving online data sources
		Ethics committees making inconsistent and delayed decisions (across jurisdictions)
	Risk of barriers hindering successful implementation or running of surveillance system	Lacking professionals adequately trained in health information technology
		Lack of security in areas of conflict
		Lacking necessary infrastructural capacity (financial, technical,governance, human resources) - especially in developing countries
		Lacking political, societal or institutional commitment
	Risk that burdens and benefits of surveillance systems are unfairly distributed	Developing countries disproportionately burdened by international surveillance effort
	FURTHER ISSUES RELATED TO SPECIFIC KINDS OF PUBLIC HEALTH SURVEILLANCE SYSTEMS	
	Risks of surveillance systems relying on genetic profiles	Surveillance activity focusing too much on genes and not enough on other potential risk factors
		Surveillance focusing on genetic profiles instead of other risk factors plays part in shifting (too much) responsibility to the individual
	Risks of real-time surveillance systems	Surveillance system influences negatively the usability of electronic medical records system other practitioners rely on
	Conflicts in running vaccine safety surveillance systems during pandemics	Conflict of prioritizing early detection of adverse events or other effectiveness-related goals in distribution of vaccines
Issues in data collection, analysis and storage	ISSUES OF PROTECTING AUTONOMY/THE RIGHT TO PRIVACY	
	Risk of people not being adequately informed about usage of their data and drop-out options – especially where data from online sources is involved	
	Risk of intentional breaches of privacy/confidentiality	Illegitimate authorities requesting data beyond what is ethically justifiable
		Individuals involved in data processing releasing data without authorisation – especially where community members are involved in verbal autopsy
	Risk of unintentional breaches of privacy/confidentiality	Unauthorised access through inappropriate storage and transfer of data – especially where digital technology is used
	Conflicts between obtaining informed consent (reflecting the values of confidentiality/ privacy/ respect for autonomy) and realizing public health benefit – especially in name- or personal-identifier-based reporting	
	RISK OF PRODUCING INADEQUATE INFORMATION TO GUIDE PUBLIC HEALTH ACTIVITIES	
	Risk of collecting data that is not sufficiently accurate or complete	Collecting incorrect/fake data from user-supplied (online) data sources
		Inadequate use of electronic collection system by professionals tainting data validity
		Software errors or manipulations of electronic collection system reducing data validity
		Collecting unrepresentative data only from parts of the population
	Risks of health professionals not passing on data for analysis	Health professionals mistrusting legitimacy, usefulness and privacy of surveillance system
		Health professionals unwilling to carry administrative costs of surveillance system (without compensation)
	Risk of inadequate analysis and interpretation of data	Gaps in evidence about subject hinder adequate interpretation
		Questionable reliability of methods used for data mining and meta-analysis
		Meticulous analysis leads to harmful delays in times of emergency
	RISK OF INADEQUATELY CONSIDERING (VULNERABLE) SUBGROUPS IN DATA COLLECTION	
	Risk of needs of (vulnerable) subgroups not becoming visible by inadequate data collection strategy	Surveillance based on online data sources excludes those without internet access
		Needs of (undocumented) migrants neglected
		Needs of the poorest neglected
		Needs of people of colour neglected
	Risk of stigmatizing subgroups by data collection strategies that target only those subgroups	Strategies particularly targeting migrants
	RISKS RELATED TO SPECIFIC DATA COLLECTION STRATEGIES	
	Risks related to using verbal autopsy for data collection	Causing emotional distress in interviewees
		Data produced from interviews not reliable
	Risks related to using anonymous unlinked blood testing for surveillance	Foregoing the possibility to inform people about disease and treatment opportunities
Issues in data reporting, sharing and using for action	ISSUES OF ADEQUATELY PROTECTING THE RIGHT TO PRIVACY/CONFIDENTIALITY IN DATA REPORTING AND SHARING	
	Risk of intentional breaches of privacy/confidentiality	Sharing data with commercial actors for their private benefit
	Risk of unintentional breaches of privacy/confidentiality	People publishing data are not adequately trained in data protection
		Publicly disclosing data ensembles that allow indirect identification of individual
		Publicly quoting social media streams
		Publicly releasing data that can be linked with other sources to identify individual
	Conflicts between protection of privacy/confidentiality and realizing public benefit in sharing data with actors outside the surveillance system	
	ISSUES OF INFLICTING HARM OR RESTRICTING FREEDOM WHEN LABELLING INDIVIDUALS/COMMUNITIES AS SUFFERING FROM HEALTH ISSUES	
	Risk of inflicting physical, social or emotional harm	Individuals experiencing psychological adverse effects
		Individuals/communities experiencing economic repercussion
		Individuals/communities experiencing stigmatization and discrimination
		Physicians rejecting difficult patients to reduce problematic situations
		Individuals not accessing the care they need to protect their privacy
	Conflicts between protection from psychosocial harm and realizing public health benefits	Protecting communities from stigmatization or benefiting them through additional resource
	Risk of restricting freedom of choice	Individuals/communities facing coercive interventions or forms of punishment
	Conflicts between not limiting individual freedom and realizing public health benefit	Implementing coercive interventions that benefit the targeted individual
		Implementing coercive interventions that benefit other individuals
	ISSUES OF FORGOING PUBLIC HEALTH BENEFITS BY NOT ADEQUATELY PUTTING DATA TO USE	
	Risk of not using data (in time) for public health action	Lacking necessary resources to act upon data
		Other political interests given priority over public health goals
	Risk of not sharing data with other actors	National protection hinders inter-governmental sharing of data
		Political interest in own visibility hinders sharing across institutions
		Insufficient resources invested in data sharing arrangement
		Incompatible processes for handling data hinder data sharing
	Risk of not adequately communicating health risks to public	Unintentionally not providing all relevant information for action
		Deliberately communicating misleading messages for political reasons
		Not finding the right level of alarm to induce adequate public reaction

**Table 3:** Conditions that make foregoing informed consent procedures (more) acceptable

Justificatory conditions	Code (can be interpreted as specification of justificatory conditions)
Effectiveness	Surveillance data is really put to use for public health purpose
Necessity	Informed consent procedures reduce data validity by introducing bias
	Less intrusive alternatives for collecting information not available
	Implementation of informed consent procedures not feasible
Least infringement	Opt-out option is provided instead
	Taking data against the will of the patient is preceded by attempt of convincing to give voluntarily (last resort)
	Minimum amount of (preferably anonymized) data necessary for surveillance purpose is collected
	All relevant information about surveillance system is supplied to people affected
	Data are maintained securely to minimize further risks
Proportionality	Benefit to be realized/harm averted through surveillance activity considerable in probability and magnitude
	Minimal Risks involved in data collection
	Implementation of informed consent procedures would demand excessive resources
	No particularly sensitive information (e.g. mental or sexual health) is collected
	Potential public health benefits outweigh considerations of privacy protection
	Implementing informed consent would set harmful standards for other surveillance programs
Public justification/engagement	The community/the public/those affected were engaged in decision.
Vulnerability^a^	Data is collected to protect the health of children (who need special protection)
	No data from children is collected (because their privacy rights need special protection)
Legitimacy^a^	Only legitimate entities trusted by the public collect surveillance data
Harm principles/unreasonable exercise requirement^a^	Surveillance activity supposed to prevent harm to other individuals, not (only) same people being surveilled

^a^Not contained in Childress et al.’s original list

### Study selection

Based on the inclusion criteria, CK and DSS screened titles and abstracts of all articles identified via PubMed independently. In case of disagreement, consensus was reached discursively. As – judging from the abstract – more or less each publication could be suspected to potentially describe ethical issues at least as quasi-incidental findings, we only included articles that explicitly addressed ethical issues (as defined above). Furthermore, CK screened the back cover descriptions and tables of content of the Google Book’s hits and excluded those not containing any relevant chapters. Among the hits were also very few journal publications that were included if relevant and if they were not duplicating the PubMed search. We then sought access via various libraries; authors of books or book chapters were also contacted directly to provide their manuscripts when we were unable to access them via our institutional libraries.

### Data analysis and synthesis

The data were analysed using an adapted version of qualitative content analysis [32]. Findings are presented as higher- and lower-level categories in a coding frame. The coding frame for ethical issues was developed inductively from the data using the strategies of progressively summarizing and subsumption [32]. Only the highest-level codes were generated deductively for which we used a life-cycle perspective, i.e., the natural life-cycle of public health surveillance activities. We assumed surveillance projects having broadly three phases: (a) design and implementation, (b) data collection and analysis, and (c) data reporting, usage and sharing. These phases correspond with the highest-level categories. An additional category “background conditions” had to be added inductively to adequately capture our findings in the literature and relates to the context in which surveillance occurs.

The framework used to analyse the arguments raised in relation to informed consent uses a similar approach. The highest-level codes were again generated deductively while the rest is based on the data. One public health ethics framework developed by Childress et al. introduces a set of “justificatory conditions” that “are intended to help determine whether promoting public health warrants overriding such values as individual liberty or justice in particular cases” [33] or in other words: to support deliberations about how to resolve situations of conflict between normative principles. We based our analysis on this particular framework since, to the best of our knowledge, they are the only ones who described justificatory conditions for conflict resolution. Those “justificatory conditions” structured the various arguments raised in the literature as highest-level categories, but had to be expanded to fit our empirical findings.

The first three authors (CK, DSS, CS) all read five articles purposefully selected (i.e., to identify as many ethical issues as possible), extracted relevant quotes, and summarized them to facilitate comparisons across our findings. CK compared the extracted quotes and paraphrases across reviewers and publications and constructed a preliminary coding frame based on integration and further summarizing of the findings (this strategy is called progressively summarizing). The draft framework, underlying interpretations, and further steps in the analysis and synthesis were discussed during a one-day workshop with four authors (CK, DSS, CS and DS) to increase validity and reliability.

For the next eleven publications, the same three authors (CK, DSS, CS) extracted relevant quotes, checked whether the existing coding frame already described the relevant issues, and introduced new categories where necessary (this strategy is called subsumption). CK integrated the findings and the results were discussed during an in-person meeting. The remaining publications were analysed by only one of the authors (CK, DSS or CS) using again the strategy of subsumption. Two further in-person meetings with all authors – one after analysis of further 32 publications and one after analysing the remaining 35 publications – were convened to help resolve any remaining coding problems, and to discuss the framework’s consistency and comprehensibility of coding formulations.

## Results

**Fig. 1:**
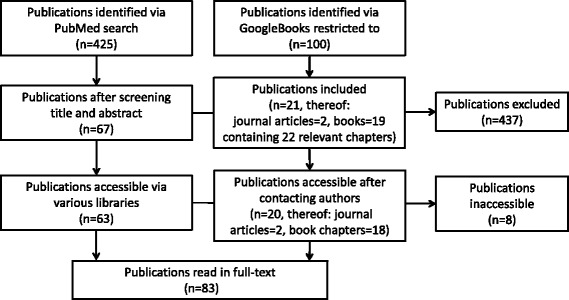
Flow diagram of literature screening process

Our literature search retrieved 525 publications of which 83 were finally included in the analysis and read in full (see Fig. 1 for screening process). Three quarter of publications were journal articles (*n* = 65, 78%) and the remaining were book chapters or whole books (*n* = 18, 22%). Articles were published between 1978 and 2014, however, the majority was published after 2000. All publications but one were written in English. A list providing bibliographical information of all 83 publications is available online (Additional file 2).

### Ethical issues

We identified 86 distinct ethical issues in public health surveillance. The main findings, per phase of the surveillance life-cycle, include:

1. *Background Conditions*: generally relate to the lack, or inappropriateness, of current guidance frameworks used for making (normative) judgements on surveillance systems. Additionally, it was discussed that certain preconditions for successful public health surveillance were not fulfilled. One example is the lack of evidence on methods employed in surveillance endangering the effectiveness of such systems.
2. *Design and Implementation Phase*: most issues relate to conflicts of priority setting with regards to the type of surveillance system to be implemented and the possibility of ill-designed systems (e.g. systems that do not adequately consider important contextual factors thereby reducing effectiveness). Furthermore, necessary surveillance systems might be inadequately or not at all implemented due to a variety of barriers (e.g. lack of funding).
3. *Data Collection and Analysis Phase*: the main issues repeatedly raised were that of privacy breaches in data collection and storage, as well as the legitimacy of foregoing informed consent. In addition, the risk of producing inappropriate data (e.g. lacking the necessary accuracy) for public health action is highlighted. Moreover, the worry is raised that certain groups might be excluded from data collection (and thereby not benefit from surveillance activity).
4. *Use of Data Phase*: issues of privacy protection are again raised, especially where data is shared with actors outside the surveillance system. Furthermore, the possibility of inadvertently inflicting harm (e.g. stigmatization or discrimination) by disseminating sensitive medical data is mentioned, as well as the risk of foregoing benefit by not putting data to adequate use.

Table 2 gives a fuller and detailed account of issues discussed.

### Justificatory conditions for (not) implementing informed consent procedures

We found 20 different conditions discussed that authors perceive to make forgoing informed consent more ethically acceptable. Again, no more than a broad overview can be provided here: It is argued that foregoing informed consent is more acceptable or justified (a) when surveillance is *effective,* e.g. which can be ensured by actually putting the data to use, (b) when it is *necessary* for public health action to forego informed consent, e.g., because otherwise data validity is endangered, (c) when the *infringement* in privacy is *minimized* by, e.g., collecting only the minimum amount of data necessary, which may include data anonymization, (d) when harms are *proportional* to benefits, e.g., where expected benefits are considerable, and (e) when the *public is engaged* in decision-making on informed consent procedures. Those arguments mirror the justificatory conditions described in the public health ethics framework our work was based on [33]. Additional codes derived inductively relate to (f) protecting *vulnerable* populations (e.g. surveillance without informed consent is acceptable to protect the health of children), (g) making sure that only *legitimate* institutions collect data, and (h) deploying the harm principle or unreasonable exercise argument, which states that collecting data from people without asking their consent might be justified where the health of other people needs protections, but should generally not be considered acceptable where the purpose is to prevent harm from the people being subject to surveillance [34]. A full overview is provided in Table 3.

In the framework presenting ethical issues we introduced three levels of codes (themes, codes, subcodes) with increasing abstraction level. It would have been possible to introduce less levels, but we found this way of presenting our findings most accessible. In tables provided as supplemental online material we furthermore present text examples for each ethical issue (Additional file 3) and justificatory condition (Additional file 4) which we will – for reasons of readability – not present here. However, this material allows retracing the process of synthesising the data.

## Discussion

Ethical issues have to be addressed in each step of implementing and running a public health surveillance system. A prerequisite for dealing with ethical issues in an adequate manner is awareness of the full spectrum and complexity of issues that will likely arise. This review therefore provides the first systematic qualitative review of the literature for those developing guidance and training material, as well as public health practitioners. As our target audience were practitioners and not (foremost) the bioethics community, we found it most helpful to use the phases of surveillance for synthesising the data and not, for example, normative principles. We could thereby ground the analysis in the practical reality of those working in the field. Our review is furthermore different from other reviews of ethical issues [18, 28] in that it also extracted proposed strategies to adequately deal with ethical issues identified, although we only present our findings on the issue of informed consent. From the standpoint of guideline developers, this broader perspective seems desirable as it allows formulating recommendations regarding how to handle diverse issues knowing all arguments put forward in the literature. This is the first study to demonstrate the feasibility of such an approach. In the future, to maximize the instrumental value for practitioners, guideline developers, and other users of analyses of this kind, systematic (qualitative) review approaches should be further refined based on feedback from users.

Generally, guidelines for conducting systematic reviews require researchers to assess the quality of the data included [26]. However, the notion of ‘quality’ is underdeveloped for ethics inquiries and we therefore refrained from any quality assessment. This also means we did not assess the practical relevance of ethical issues – one promising candidate for operationalizing quality in this context. The number of articles devoted to one issue does not function as a reliable indicator of importance due a number of potential limiting factors (e.g. publication bias), which was also the reason why we did not count the number of times a particular code was discussed in the literature. Moreover, we did not address the normative relevance of justificatory conditions introduced in the literature. Decision-makers will have to weigh the different arguments within and for their specific contexts.

Nevertheless, we want to raise a few comments with regards to the quality and breadth of the scientific discourse in the field of public health surveillance. First, we found it critical that most theoretical articles we read did not substantiate real-life risks described with empirical data that could have demonstrated their relevance. To give but one example: stigmatization as a result of surveillance is of course a theoretical possibility, but it is hardly discussed how often, in what setting, or for which diseases stigmatization and discrimination are actually experienced as a consequence of published surveillance data. Judging from the literature we have read, the relevant empirical literature is not adequately taken into account in normative documents though authors’ normative arguments often rested on empirical premises. Normative scholars in public health should therefore make a point of considering the relevant empirical literature more explicitly.

It was furthermore noteworthy that among the publications included only one focused specifically on a low- income country [35] and two focused on a method of data collection (verbal autopsy) that is generally only employed in a low-income context [36, 37]. Furthermore, five papers focused on global public health surveillance, which actually included the low- and middle-income countries’ perspective [38–42]. However, the majority of articles focused on privacy in digitalized surveillance systems, which may be of greater importance in high-income countries. It seems that the perspective of low-income countries is not well represented in the literature possibly due to preferences of researchers, funding opportunities or publication bias. However, we do not think that this is particular for the field of surveillance research, but rather represents a general imbalance in knowledge generation that tends to focus on financially strong contexts. As to be expected, there are no ready-made solutions for this problem.

Regarding the discussion of justificatory conditions, we found it questionable that many articles stayed on a very abstract level. Often authors just restated the – rather abstract and broad – justificatory conditions already outlined in the framework of Childress et al. [33]. It is not enough to just say that the benefits of surveillance need to be considerable to justify foregoing informed consent, practitioners need to know what would constitute a (in-) considerable benefit. It would be desirable if more authors could like Lee et al. [5] at least provide examples (in her case HIV where limited data could lead to underfunding of programs, increase infections and ultimately death or so she claims) and rationales for why certain justificatory conditions are fulfilled in a particular context. Ethicists motivated to improve public health practice should not shy away from more context-specific analyses, thereby also providing example cases of normative deliberations that practitioners will inevitably have to address.

Lastly, we want to emphasize again that systematic reviews can only be a starting point for any policy process. Other types of ethical inquiry – like commissioned evaluation of arguments or analysis of ethical issues in novel areas – might and possibly should supplement our research as information base for decision-making. Given that the literature will always be limited due to personal preferences, funding priorities and scientific paradigms, it has been recommended [20, 43] that systematic review findings are accompanied by hearings or surveys of experts and possibly affected communities. This will ensure that all important issues and arguments are captured and discussed during guideline or further policy development.

### Limitations

One limitation of this review is the inclusion of only two databases (PubMed and Google Books). However, those can be considered the most important ones, allowing us to include both books and journal articles. Previous systematic reviews of medical ethics literature have shown only marginal additional value of searching other databases like EMBASE or Euroethics [19, 44]. We could have also included additional terms to our search strategy, for example, data sources for surveillance like “screening” or “health surveys”. However, this would have increased the number of hits for us to screen considerably while promising only few additional relevant findings. Furthermore we did not have to adapt our matrix on the level of themes and only had to make marginal changes on the level of codes for the last round of analysis of the final 35 publications. We therefore assumed thematic or conceptual saturation for the level of codes, meaning that analyzing further documents would not reveal other ethical issues at the abstraction level of codes [45]. Due to language capacities within the research team we could only include articles published in English, German, and Spanish. However, only two papers (one written in French [46] and one in Portuguese [47]) were excluded based on language. We are therefore confident that we have captured the majority of issues.

One could criticize how we operationalized the fundamental but contested concepts that grounded our search, first and foremost how we defined what constitutes an ethical issue. First, we based our approach on principlism. This lens might have complicated capturing more postmodern or constructivist discussions and some issues that can arguably be described as ethical will not have been identified here. One example would be discussions that try to clarify a particular concept – e.g. privacy rights and their normative justification – or discussions that do not refer to e.g. harm or autonomy violations, but more diffuse notions of the good to criticize certain actions. However, we had good reasons for choosing an approach based on principlism. Most importantly, other systematic qualitative reviews of normative information already demonstrated the instrumental value of this approach for a descriptive and stakeholder-oriented analysis and synthesis of normative arguments [18, 19, 28]. Additionally, we found this approach to be more grounded in the actual theoretical discussions in the public health ethics literature than, for example, an approach relying on the authors’ statement in the abstract to discuss ethical issues (which other reviews relied on [48]). Second, we also chose one particular principlist framework over possible alternatives [29]. It is difficult to say how choice of a different framework might have impacted our analysis, but we are convinced that the inclusive nature of the chosen framework with regards to principles translated to inclusiveness of our research with regards to issues and arguments. Further research still has to demonstrate whether alternative approaches result in equal, superior, or inferior results.

Additionally, systematic qualitative ethics reviews involve a high level of interpretation because authors do not always clearly describe the issues at hand, thereby introducing a subjective or interpretive element to the analysis on the part of those conducting the review. However, three authors were involved in reading and analysing the literature and met regularly to discuss challenges with interpretations to ensure the reliability and validity of the findings. We are therefore confident that our interpretations properly represent the data.

## Conclusion

This article gives a comprehensive overview of ethical issues in public health surveillance discussed in the literature. The main findings of the review were of crucial importance to the development of WHO’s guidelines. While specifically helpful and important for development of guidance documents, it can function as an information base for various actors and can also inform the development of policies and training materials. However, we want to stress that just because this review is conducted in a systematic and transparent fashion, further steps will not automatically follow. There need to be processes in place to ensure that the issues to be addressed in a guidance policy document are carefully chosen and that recommendations are formulated in an iterative manner taking into account many expert voices and, preferably, of those persons ultimately affected by a particular policy.

Assuming that systematic (qualitative) reviews will play an increasingly important role in ethics guidance development, it will be of importance to introduce feedback loops between reviewers and guideline developers. Thereby the methodology of systematic ethics reviews can continuously be improved and adjusted to the specific contextual needs. The same holds true for other information used in guideline development.

## Additional files

Additional file 1: PRISMA checklist (completed). (PDF 67kb)

Additional file 2: Papers read in full text. (PDF 28kb)

Additional file 3: Ethical issues in public health surveillance with example quotes. (PDF 373kb)

Additional file 4: Conditions that make foregoing informed consent procedures (more) acceptable with example quotes. (PDF 290kb)

## Footnotes

1 We do not want to imply that those types of articles are problematic. In fact we believe that context- or issue-specific analysis can serve a vital function in informing practitioners. The articles published so far were, however, not sufficient as evidence base for the development of guidelines where comprehensiveness and systematic reduction of bias is attempted.

2 We are aware that the term risk is generally used to denote harm potential, however, this is not how it is used here. With the introduction of the above definition we are not making any conceptual claims with regards to risk, but introduced the terms risk and conflict pragmatically to delineate the two different types of ethical issues.
