# Supplementary material for: Ethical issues in public health surveillance: a systematic qualitative review
Source: BMC Public Health. 2017 Apr 4;17:295. doi: 10.1186/s12889-017-4200-4 (PMC5381137; doi:10.1186/s12889-017-4200-4)
Supplement: Supplementary file 2 — Papers read in full text. (PDF 28kb) [file 12889_2017_4200_MOESM2_ESM.pdf]

PAPERS READ IN FULL TEXT

Journal articles

1. Anoshiravani A, Gaskin GL, Groshek MR, Kuelbs C, Longhurst CA. Special requirements for electronic medical records in adolescent medicine. *J Adolesc Health.* 2012;51(5):409-414.
2. Bank R, Laska EM. Protecting privacy and confidentiality in a multiple use, multiple user mental health information system. *Eval Program Plann.* 1978;1(2):151-157.
3. Barnett T, Sorenson C. Infectious disease surveillance in the United States and the United Kingdom: from public goods to the challenges of new technologies. *J Health Polit Policy Law.* 2011;36(1):165-185.
4. Bauer S. Societal and ethical issues in human biomonitoring--a view from science studies. *Environ Health.* 2008;7 Suppl 1:S10. doi: 10.1186/1476-069X-7-S1-S10.
5. Bayer R, Fairchild AL. Public health. Surveillance and privacy. *Science.* 2000;290(5498):1898-1899.
6. Bayer R, Greco DB, Ramachandran R. The ethics of clinical and epidemiological research. *Int J Tuberc Lung Dis.* 2011;15 Suppl 2:S25-29. doi: 10.5588/ijtld.10.0618.
7. Bernstein AB, Sweeney MH. Public health surveillance data: legal, policy, ethical, regulatory, and practical issues. *MMWR Suppl.* 2012;61(3):30-34.
8. Briggs CL, Nichter M. Biocommunicability and the biopolitics of pandemic threats. *Med Anthropol.* 2009;28(3):189-198.
9. Brody JG, Dunagan SC, Morello-Frosch R, Brown P, Patton S, Rudel RA. Reporting individual results for biomonitoring and environmental exposures: lessons learned from environmental communication case studies. *Environ Health.* 2014;13:40. doi: 10.1186/1476-069X-13-40.
10. Brooks JI, Sandstrom PA. The power and pitfalls of HIV phylogenetics in public health. *Can J Public Health.* 2013;104(4):e348-350.
11. Calain P. From the field side of the binoculars: a different view on global public health surveillance. *Health Policy Plan.* 2007;22(1):13-20.
12. Chandramohan D, Soleman N, Shibuya K, Porter J. Ethical issues in the application of verbal autopsies in mortality surveillance systems. *Trop Med Int Health.* 2005;10(11):1087-1089.

13. Chretien JP, Yingst SL, Thompson D. Building public health capacity in Afghanistan to implement the International Health Regulations: a role for security forces. *Biosecur Bioterror.* 2010;8(3):277-285.
14. Clarke C, Magnusson RS. Data registers in respiratory medicine: a pilot project evaluating compliance with privacy laws and the National Statement on Ethical Conduct in Research Involving Humans. *J Law Med.* 2002;10(1):69-83.
15. Cloud D, Dougherty M, May RL, 2nd, Parsons J, Wormeli P, Rudman WJ. At the intersection of health and justice. *Perspect Health Inf Manag.* 2014;11:1c. eCollection 2014.
16. Conway M. Ethical issues in using Twitter for public health surveillance and research: developing a taxonomy of ethical concepts from the research literature. *J Med Internet Res.* 2014;16(12):e290. doi: 10.2196/jmir.3617.
17. Coughlin SS. Ethical issues in epidemiologic research and public health practice. *Emerg Themes Epidemiol.* 2006;3:16.
18. Cushman R. Serious technology assessment for health care information technology. *J Am Med Inform Assoc.* 1997;4(4):259-265.
19. Datta J, Wellings K, Kessel A. 'Once the stuff's left my body, it's not me': service users' views on unlinked anonymous testing of blood for HIV. *Cult Health Sex.* 2013;15(8):896-909.
20. Dhopeshwarkar RV, Kern LM, O'Donnell HC, Edwards AM, Kaushal R. Health care consumers' preferences around health information exchange. *Ann Fam Med.* 2012;10(5):428-434.
21. Dumez B, Van Damme K, Casteleyn L. Research on ethics in two large Human Biomonitoring projects ECNIS and NewGeneris: a bottom up approach. *Environ Health.* 2008;7 Suppl 1:S7. doi: 10.1186/1476-069X-7-S1-S7.
22. Eisenstein M. Public health: An injection of trust. *Nature.* 2014;507(7490):S17-19.
23. Eng TR. Population health technologies: emerging innovations for the health of the public. *Am J Prev Med.* 2004;26(3):237-242.
24. Evans B, Ramsay CN. Using patient identifiable data without consent. Integrity of communicable disease surveillance is important patient care. *BMJ.* 2001;322(7290):859.
25. Fairchild AL. Dealing with Humpty Dumpty: research, practice, and the ethics of public health surveillance. *J Law Med Ethics.* 2003;31(4):615-623.

26. Fairchild AL, Alkon A. Back to the future? Diabetes, HIV, and the boundaries of public health. *J Health Polit Policy Law.* 2007;32(4):561-593.
27. Fairchild AL, Bayer R. Public health. Ethics and the conduct of public health surveillance. *Science.* 2004;303(5658):631-632.
28. Fairchild AL, Gable L, Gostin LO, Bayer R, Sweeney P, Janssen RS. Public Goods, Private Data: HIV and the History, Ethics, and Uses of Identifiable Public Health Information. *Public Health Rep.* 2007;122(Suppl 1):7-15.
29. Fairweather NB, Rogerson S. A moral approach to electronic patient records. *Med Inform Internet Med.* 2001;26(3):219-234.
30. Fleming DM. Wanted: social contract for practice of medicine. Ethical framework for using medical records is needed. *BMJ.* 2001;323(7318):930.
31. Goldman LR, Anton-Culver H, Kharrazi M, Blake E. Banking of human tissue for biomonitoring and exposure assessment: utility for environmental epidemiology and surveillance. *Environ Health Perspect.* 1995;103 Suppl 3:31-34.
32. Goodman KW. Ethics, information technology, and public health: new challenges for the clinician-patient relationship. *J Law Med Ethics.* 2010;38(1):58-63.
33. Gostin LO. Law and ethics in population health. *Aust N Z J Public Health.* 2004;28(1):7-12.
34. Henriksen E, Johansen MA, Baardsgaard A, Bellika JG. Threats to information security of real-time disease surveillance systems. *Stud Health Technol Inform.* 2009;150:710-714.
35. Hiratsuka V, Brown J, Dillard D. Views of biobanking research among Alaska native people: the role of community context. *Prog Community Health Partnersh.* 2012;6(2):131-139.
36. Hodge JG, Jr., Gostin LO, Jacobson PD. Legal issues concerning electronic health information: privacy, quality, and liability. *JAMA.* 1999;282(15):1466-1471.
37. Iskander J, Haber P, Herrera G. Monitoring vaccine safety during an influenza pandemic. *Yale J Biol Med.* 2005;78(5):265-275.
38. Kehr J. Blind spots and adverse conditions of care: screening migrants for tuberculosis in France and Germany. *Sociol Health Illn.* 2012;34(2):251-265.
39. Kotecha JA, Manca D, Lambert-Lanning A, et al. Ethics and privacy issues of a practice-based surveillance system: need for a national-level institutional research ethics board and consent standards. *Can Fam Physician.* 2011;57(10):1165-1173.

40. Lee LM, Heilig CM, White A. Ethical justification for conducting public health surveillance without patient consent. *Am J Public Health.* 2012;102(1):38-44.
41. Lopez YL, Gonzalez C, Gallego BN, Moreno AL. [Stewardship of public health surveillance in the health system in Colombia: a case study]. *Biomedica.* 2009;29(4):567-581.
42. Master Z, Claudio JO, Rachul C, Wang JC, Minden MD, Caulfield T. Cancer patient perceptions on the ethical and legal issues related to biobanking. *BMC Med Genomics.* 2013;6:8. doi: 10.1186/1755-8794-6-8.
43. Magnusson RS. Testing for HIV without specific consent: a short review. *Aust N Z J Public Health.* 1996;20(1):57-60.
44. Magnusson RS. Data linkage, health research and privacy: regulating data flows in Australia's health information system. *Syd Law Rev.* 2002;24(1):5-55.
45. McNabb SJ. Comprehensive effective and efficient global public health surveillance. *BMC Public Health.* 2010;10 Suppl 1:S3. doi: 10.1186/1471-2458-10-S1-S3.
46. Middaugh JP, Hodge JG, Cartter ML. The ethics of public health surveillance. Comment on Public Health. Ethics and the conduct of public health surveillance. *Science.* 2004;304(5671):681-684.
47. Mony PK, Vaz M. A qualitative inquiry into the application of verbal autopsy for a mortality surveillance system in a rural community of southern India. *World Health Popul.* 2011;13(1):30-39.
48. Ng ES, Tambyah PA. The ethics of responding to a novel pandemic. *Ann Acad Med Singapore.* 2011;40(1):30-35.
49. O'Dowd A. Medical data: does patient privacy trump access for research? *BMJ.* 2013;347:f5516. doi: 10.1136/bmj.f5516.
50. Petrini C. Ethics in public health surveillance. *Ann Ist Super Sanita.* 2013;49(4):347-353.
51. Petrini C. Procedures for the ethical review of public health surveillance protocols. Commentary. *Ann Ist Super Sanita.* 2014;50(1):1-3.
52. Ploug T, Holm S. Pharmaceutical information systems and possible implementations of informed consent -- developing an heuristic. *BMC Med Ethics.* 2012;13:30. doi: 10.1186/1472-6939-13-30.
53. Resnik DB. Responsibility for health: personal, social, and environmental. *J Med Ethics.* 2007;33(8):444-445.

54. Richards EP. Dangerous people, unsafe conditions. The constitutional basis for public health surveillance. *J Leg Med.* 2009;30(1):27-50.
55. Rollin BE. Veterinary medical ethics. An ethicist's commentary on the case of the client who abuses disease surveillance results. *Can Vet J.* 2011;52(2):113-114.
56. Rubel A. Justifying public health surveillance: basic interests, unreasonable exercise, and privacy. *Kennedy Inst Ethics J.* 2012;22(1):1-33.
57. Santos RP, Mayo TW, Siegel JD. Healthcare epidemiology: active surveillance cultures and contact precautions for control of multidrug-resistant organisms: ethical considerations. *Clin Infect Dis.* 2008;47(1):110-116.
58. Sengupta S, Calman NS, Hripcsak G. A model for expanded public health reporting in the context of HIPAA. *J Am Med Inform Assoc.* 2008;15(5):569-574.
59. Sturtevant JL, Anema A, Brownstein JS. The new International Health Regulations: considerations for global public health surveillance. *Disaster Med Public Health Prep.* 2007;1(2):117-121.
60. Taylor CE. Surveillance for equity in primary health care: policy implications from international experience. *Int J Epidemiol.* 1992;21(6):1043-1049.
61. Teo P, Yeoh BS, Ong SN. SARS in Singapore: surveillance strategies in a globalising city. *Health Policy.* 2005;72(3):279-291.
62. Turnberg L. Common sense and common consent in communicable disease surveillance. *J Med Ethics.* 2003;29(1):27-29.
63. Vayena E, Mastroianni A, Kahn J. Ethical issues in health research with novel online sources. *Am J Public Health.* 2012;102(12):2225-2230.
64. Verity C, Nicoll A. Consent, confidentiality, and the threat to public health surveillance. *BMJ.* 2002;324(7347):1210-1213.
65. Wurtz R. The role of public health in health information exchanges. *J Public Health Manag Pract.* 2013;19(5):485-487.

Books and book chapters:

1. Blatt AJ. Data Privacy and Ethical Uses of Volunteered Geographical Information. In: Blatt AJ. *Health, Science, and Place - A New Model.* Dordrecht: Springer; 2015: 49-59.
2. Childress JF. Surveillance and Public Health Data: The Foundation and Eyes of Public Health. In: Bernheim RG, Childress JF, Bonnie RJ, Melnick AL, eds. *Essentials of Public Health Ethics*. Burlington: Jones & Bartlett Learning; 2015:97-118.

3. Collmann J, Robinson A. Designing Ethical Practice in Biosurveillance. In: Zeng D, Chen H, Castillo-Chavez C, Lober WB, Thurmond M, eds. *Infectious Disease Informatics and Biosurveillance*. Dordrecht: Springer; 2011:23-44.
4. Datta J, Kessell A. Unlinked anonymous blood testing for public health purposes: an ethical dilemma? In: Peckham S, Hann A, eds. *Public Health Ethics and Practice*. Bristol: Policy Press; 2010:101-116.
5. Fairchild AL, Bayer R, Colgrove J. *Searching Eyes: Privacy, the State, and Disease Surveillance in America.* Berkeley: University of California Press; 2007.
6. Fairchild AL, Jones MM. Ethics and the conduct of public health surveillance. In: M´ikanatha NM, Lyfield R, Van Beneden CA, de Valk H, eds. *Infectious Disease Surveillance*. Malden: Blackwell Publishing; 2007:445-449.
7. Gilbert GL. Electronic Surveillance for Communicable Disease Prevention and Control: Health Protection or a Threat to Privacy and Autonomy? In: Enemark C, Selgelid MJ, eds. *Ethics and Security Aspects of Infectious Disease Control: Interdisciplinary Perspectives*. Surrey: Ashgate; 2012:127-143.
8. Heilig CM, Sweeney P. Ethics in Public Health Surveillance. In: Lee LM, Teutsch SM, Thacker SB, St. Louis ME, eds. *Principles & Practice of Public Health Surveillance*. 3rd ed. Oxford: Oxford University PRess; 2010: 198-216.
9. Lee LM. Extending Ethical Justification for Public Health Surveillance to Situational Awareness. In: Enemark C, Selgelid MJ, eds. *Ethics and Security Aspects of Infectious Disease Control: Interdisciplinary Perspectives*. Surrey: Ashgate; 2012:111-126.
10. Lee LM. Health Information in the Background: Justifying Public Health Surveillance Without Patient Consent. In: Pimple KD, ed. *Emerging Pervasive Information and Communication Technology (PICT): Ethical Challenges, Opportunities and Safeguards*. Dordrecht: Springer; 2014:39-53.
11. Goodman K, Meslin E. Ethics, Information Technology, and Public Health: Duties and Challenges in Computational Epidemiology. In: Magnuson JA, Fu JPC, eds. *Public Health Informatics and Information Systems*. London: Springer; 2014:191-209.
12. Gostin LO. Surveillance and Public Health Research: Privacy and the "Right to Know". In: Gostin LO, ed. *Public Health Law and Ethics: A Reader*. Berkeley: University of California Press; 2002:295-333.
13. Poudrier J. "Racial" Categories and Health Risks: Epidemiological Surveillance. In: Lyon D, ed. *Surveillance as Social Sorting: Privacy, Risk, and Automated Discrimination*. Abingdon: Routledge; 2003:111-134.

14. Rhodes R, Baumrin B, Earle WJ, et al. Public Health and Research on Populations. In: Rhodes R, Gligorov N, Schwab AP, eds. *The Human Microbiome: Ethical, legal and social concerns*. Oxford: Oxford University Press; 2013:208-246.
15. Snider DE, Stroup DF. Ethical Issues. In: Teutsch SM, Churchill RE, eds. *Principles and Practice of Public Health Surveillance*. 2nd ed. Oxford: Oxford University Press; 2000:194-214.
16. Szczepaniak MC, Wagner MM, Hutman J, Daswani S, Goodman KW. Advancing Organizational Integration: Negotiation, Data Use Agreements, Law and Ethics. In: Wagner MM, Moore AW, Aryel RM, eds. *Handbook of Biosurveillance*. Burlington: Elsevier Academic Press; 2006:465-480.
17. van der Ploeg I. Biometrics, and the body as information: normative issues of the socio-technical coding of the body. In: Lyon D, ed. *Surveillance as Social Sorting: Privacy, Risk, and Automated Discrimination*. Abingdon: Routledge; 2003:57-74.
18. Yasnoff WA. Privacy, Confidentiality, and Security of Public Health Information. In: Magnuson JA, Fu JPC, eds. *Public Health Informatics and Information Systems*. London: Springer; 2014:155-172.
