# Supplementary material for: Ethical issues in public health surveillance: a systematic qualitative review
Source: BMC Public Health. 2017 Apr 4;17:295. doi: 10.1186/s12889-017-4200-4 (PMC5381137; doi:10.1186/s12889-017-4200-4)
Supplement: Supplementary file 3 — Ethical issues in public health surveillance with example quotes. (PDF 373kb) [file 12889_2017_4200_MOESM3_ESM.pdf]

Ethical issues in public health surveillance with example quotes

Field	Themes		
	**Code**	**Subcode**	**Example quote**
Background issues	Issues related to choice of framework for conducting public health surveillance		
	Risk of misguided judgement due to lacking ethical framework	Lacking ethical framework for using online data sources	“More recently, the public health community has recognized Twitter’s potential for public health surveillance with applications including monitoring the prevalence of infectious diseases in the community, identifying early-stage disease outbreaks, detecting disease outbreaks in mass gatherings, and recognizing and understanding health behaviors, like temporal variability in problem drinking, and attitudes toward emerging tobacco products such as electronic cigarettes and hookah. Despite the clear utility of using Twitter to augment current public health surveillance, there remains doubt among regulatory authorities, ethics committees, and individual researchers regarding ethically appropriate conduct in this kind of large-scale research, where a single researcher can automatically process hundreds of millions of public tweets.” (Conway 2014)
		Lacking ethical framework for how to treat data of the deceased	„There has been remarkably little consideration of moral obligations with respect to the dead, but the issue is in practice inescapable when considering electronic patient records: a high proportion of the entries on an EPR are likely to relate to the period immediately before death.” (Fairweather & Rogerson 2001)
	Risk of misguided judgement due to using inappropriate ethical framework	Using research ethics framework (because criteria for differentiating research and surveillance are missing)	“When theses routine practices become a form of population-based research is a vexing and important problem, because if routine public health practices are classified as “research”, health departments would have to submit this activity for review by institutional review boards (IRBs) and obtain informed consent from participants. Classification of practice as research, therefore, could impede rapid and effective responses to community health threats. In short, when public health surveillance is classified as public health research, it could entail both formal IRB review and informed consent by participants, which could cause risky delays in generating valuable information.” (Childress 2015)
		Employing the research vs. practice paradigm	„The deeper problem is that whether an activity is research or practice tells us nothing whatever about what actions are justified as part of that activity. That

		that lacks moral salience	is, the distinction between research and practice has no independent moral importance. And the mere fact that something is categorized as practice rather than research fails to provide a reason why the activity should be exempt from IRB oversight and privacy protections such as those afforded under the Common Rule." (Rubel 2012)
		Using clinical ethics framework	"HBM studies often require a maximal participation of given populations in order to provide reliable knowledge, which may then be beneficial for protecting people through preventive action. The appropriateness of the current legal and ethical framework, mainly issuing from and applicable to clinical medicine, and therefore representing a strongly individual-oriented approach, should therefore be assessed." (Dumez et al. 2008)
		Using health security framework	"When public health is considered a problem of health security, and when the war against disease is connected with the war against human enemies, surveillance becomes even more important. One result may be that is has become easier to accept and justify public health surveillance without close attention to the relevant ethical considerations, to which we now turn." (Childress 2015)
	Issues related to scientific standards for evidence generation	Conflict between different knowledge systems	"What's at stake here? Discursive practices are clearly changing. Although state, international, and corporate biosecurity regimes are increasingly defining and regulating knowledge about infectious diseases, many among us are willing to explore alternatives. Any account of infectious disease reifies certain players, circuits, practices, and forms of authority and imbues them with different moral and affective characters to produce and hold knowledge. In the process, competing perspectives are erased or at least subordinated as unsubstantiated beliefs. When we ask ''Is this real or is it overblown?'' what seems to be a grab for agency signals engagement with complex notions of subjectivity, authority, knowledge, intertextuality, space, time, and knowledge=action relations. So maybe it's time to ask a different set of questions." (Briggs & Nichter 2009)
		Risk of choosing framework for evidence generation that hinders production and use of	"In view of the ethical issues arising in prospective observational studies that would evaluate preventive interventions, the implications of the very frameworks for evidence and proof for science and policy action need to be discussed; this cannot be resolved by science alone; they require a broad

		relevant data	debate on the conditions of proof requested for action on a societal level. To account for fields where no RCTs are possible, a protocol for observational studies has been developed, similar to the idea of the Cochrane collaboration; for environmental health, these evidence criteria require further reflection and elaboration. The perceptibility of environmental health issues will depend on how the concept of evidence is framed and on how the burden of proof for environmental risks is distributed in society.“ (Bauer 2008)
	Risk of not fulfilling preconditions for successful public health surveillance		
	Risk of barriers hindering development of technology to improve effectiveness and efficiency of surveillance	Lacking funding for technology development	“To date, few investors and technology developers have paid attention to population health–oriented products compared with individually oriented, medical care interventions (e.g., diagnostics and treatment modalities). This emphasis is reflected in the fact that the bulk of the health care expenditures in the United States—$1.3 trillion in 2000—is spent on medical care interventions. Although no official data are collected on PHT [*comment by author: population health technology*] expenditures, such investment is likely insignificant compared with support for medical technology research and development (R&D). For example, pharmaceutical companies belonging to a major industry trade group invested more than $30 billion in R&D in 2001; this investment does not include expenditures by other medical care industries, such as device and equipment manufacturers. There is no major public or private funding program for PHT R&D to my knowledge.” (Eng 2004)
		Lacking necessary multidisciplinary collaboration for technology development	“In addition, the markets for PHTs have not been well defined, and there is a lack of professional and public understanding of this nascent field. Whereas there is a strong track record of technology transfer among disciplines like computer science, commercialization of ideas from population health–related institutions, such as schools of public health, is rare because they lack the technology or business expertise to develop or commercialize technologies. Similarly, technologists and entrepreneurs typically do not have the necessary expertise in population health and research. Development of PHTs requires a multidisciplinary and multisector approach that involves stakeholders who do not usually communicate or collaborate with each other.” (Eng 2004)
	Risk of not producing sufficiently robust evidence on		„It is generally assumed that all communicable disease surveillance systems set up in industrialized countries perform optimally, but evidence is lacking.

	effective surveillance methods		Systematic assessments of surveillance systems by external evaluators have targeted mainly developing or transitional countries, leaving some questions open as to what is the standard 'reference' performance in public health surveillance." (Calain 2006)
Issues in system design and implementation	Issues of deciding which public health surveillance system should be realized		
	Conflicts of priority setting between different public health programs	Prioritizing between different public health surveillance systems	"Current best practices for the 7 activities within the 3 basic steps of a public health surveillance system begin with system development, which includes beginning with a clear understanding of the public health purpose of the system to ensure that the applicable data are collected to answer the key questions. A clear understanding of what public health question to answer often involves prioritizing among health outcomes on the basis of the magnitude of the effect, measured by frequency, severity, cost, or preventability." (Lee et al. 2012)
		Prioritizing between surveillance activity and other public health activities	„An equally successful achievement is about to be reached in the coming years with polio eradication, through the international surveillance of acute flaccid paralysis (AFP). However efficient and appropriate they may be, these 'categorical' interventions carry a price to pay at the expense of public health services." (Calain 2006)
		Prioritizing potential emerging threats or sustained health issues	"We all know that the worst can happen and the SARS epidemic of 2003 has been a warning signal, now superseded by the widespread epizootic expansion of avian (H5N1) influenza. But to what extent should alarms distract policy-makers from sustained and blatant emergencies, such as major endemic diseases, the breakdown of health systems or the much neglected shortage of local professional skills?" (Calain 2006)
	Risk of wasting resources by prioritizing surveillance systems	Prioritizing disease areas important for developed nations instead of areas of high need	"Developing nations may be pressured to improve their surveillance infrastructure for novel potential pandemic agents, but in doing so may divert resources from areas of greater need, such as endemic conditions like Acquired Immunodeficiency Syndrome (AIDS), tuberculosis and malaria. The current H1N1 2009 pandemic (for which WHO has finally declared the post-pandemic phase) claimed 18,000 lives in the year and two months since its appearance, approximately the number of individuals who die in one week from malaria." (Ng &Tambyah 2011)
		Prioritizing surveillance	„While considerable resources and attention were now being directed toward

		systems where other investments would serve public health better	infectious diseases, the United States and United Kingdom (along with other major donors) were criticized for focusing their investments and activities in infectious disease control, and global health more broadly, on specific diseases or through vertical programs, rather than on the complex, myriad health system, economic, social, political, and cultural factors underlying disease risks.“ (Barnett & Sorenson 2011)
	Issues of adequately designing a public health surveillance system		
	Conflicts of priority setting within the design of a surveillance program	Prioritizing comprehensiveness and accuracy of data or efficiency of surveillance system	“The advantage of active surveillance by public health officials is that it tends to be more comprehensive and its data tend to be more complete; the disadvantage is that it requires more resources than passive surveillance.” (Childress 2015)
		Prioritizing efficiency by minimizing costs or security of data protection when employing digital technology	“Due to considerations such as the principle of beneficence, in healthcare there is a need to cut costs that are not inevitable costs of treatment where this can be done without harming treatment. Electronic transfer of patient records offers the potential to save money when compared to traditional methods, freeing resources for ‘front-line’ patient care. As all security and privacy technologies come with associated costs, there is a direct tension here between privacy and financial goals.” (Fairweather & Rogerson 2001)
		Prioritizing early detection of events or efficiency trough reduction of false-positive alarms	"The earlier the event a system monitors, the earlier public health can use the information to guide decisions. However, the earlier the system monitors a condition, the more likely it is to capture "false positives" or to identify someone who, initially identified as a case, will not progress to the diagnosis of interest." (Lee 2012)
		Prioritizing maximizing amount and utility of data or security of private information by limiting data collected	"Ideally it should be impossible for any individual in the database to be identified. However, this limits the amount of information that can be collected, making it impossible to conduct validated health surveillance. There is always a trade- off between utility and security, therefore, and a small risk of identification." (Kotecha et al. 2011)
		Prioritizing harmonization of methods to improve	"The Office of Management and Budget mandates the use of specific questions for selected variables (e.g., race, ethnicity, and sex). This is a first step in promoting standards for data that can be used in public health surveillance.

		sharing arrangements or tailoring to specific purpose	The next steps are to standardize data formats and data elements, codes, and methods across programs to meet the needs of both data collectors and surveillance programs. However, some flexibility must be maintained to ensure collection of the most accurate and appropriate data to meet the goals of the surveillance systems." (Bernstein & Sweeney 2012)
	Risk of making poor choices in design of the surveillance system	Not adequately considering equity issues in surveillance system	Second is the issue of environmental justice, often referred to as environmental equity. [...] It will be very important for any national program of adipose tissue banking, like any program of environmental surveillance, to over-sample in low income and various racial and ethnic communities. If past studies can serve as a guide, we can expect that we will be more likely to find higher exposures in these communities. Researchers, who rarely are from these areas, should not assume that they understand the needs for these communities or the consequences of their findings for the residents. Certainly, there is likely to be less access to education, poorer nutrition in low income areas, and cultural differences in understanding of health and disease. Such communities are also less likely than a white suburban community to have resident experts such as scientists, physicians, attorneys, and other trained professionals who can interpret the data for neighbors; and they have less access to such expertise overall. In consequence, these communities may have more difficulty in taking steps needed to deal with any problems that might be identified, or compelling the government to do so." (Goldman et al. 1995)
		Not adequately tailored to the purpose and context of surveillance	"When it comes to the development of targeted screening policies of population subgroups most at risk for disease, the national surveillance reports are therefore insufficient to inform public health practice, despite their political mission to do so. Rewritten in the same format with very similar expressions every year, the German and French surveillance reports based on mandatory declarations are, I would argue, a bureaucratic form of epidemiology but not an effective and practical means to inform prevention programmes, as their mission statements hold." (Kehr 2012)<br><br>"Here, input harmonisation has been understood as somewhat ideal data recording system in a common political space, where data are collected in identical formats. However this wouldn't allow for regionally specific studies,

			because the context- specific variables would no longer be available and locally meaningful research may be precluded." (Bauer 2008)
		Not employing health information technology and other promising tools for improvement of surveillance activity	"Since adoption of this technology is moving forward with good reason anyway for clinical purposes, it would be a missed opportunity not to use the same technology for public health. It has been our thesis here that this technology bids fair to improve public health in nontrivial ways, an empirical claim; and that clinicians and patients therefore share a moral obligation to support such improvement, an ethical claim." (Goodman 2010)
		Not adequately coordinating and integrating surveillance initiatives with other services – especially in developing countries	"In a context where the acute shortage of skilled human resources and the great difficulties of the curative sector will remain unsolved for years, it is difficult to imagine how the multiplication of parallel and poorly coordinated surveillance initiatives, and their targeted funding, will achieve much more than consolidating a fragmented, inefficient and disruptive donor-driven surveillance industry." (Calain 2007)
		Not involving communities in development and implementation of surveillance systems	"Public engagement includes providing information to the public and justifying surveillance activities in light of public values. However, in its ideal form, it is more than a one way activity, with public health officials providing information and reasons to the public. Ideally it would include substantial public input, in part because public trust and cooperation are essential. In short, there are strong reasons for vigorous public engagement, with all potential stakeholders - both professionals and members of the public - in the process of developing and implementing surveillance policies. Such public engagement has become even more urgent in the context of concerns about health security and securitization." (Childress 2015)
		Commissioning actors that work ineffectively, inefficiently or unethically with running of surveillance system	"In other situations, an agency or program receives funds to collect and/or analyze specific data when it would be more efficient, or effective, for another program to do so. The funding streams and mechanisms affect how data are collected." (Bernstein & Sweeney 2012)
		Setting up surveillance systems that are inherently unsustainable, unreliable or insensitive	"Rumour surveillance is meaningful only when adequate human resources can be allocated to the systematic collection, verification and analysis of unofficial information, most of which will lead to false alerts. This might be problematic in developing countries where ministry staff are already overburdened with the

		(without adequate safeguards in place)	management of official surveillance reports." (Calain 2007)<br><br>"The IHR does not tell nations how to conduct surveillance but rather tells them what results surveillance should produce. Although this offers national governments a great deal of freedom to determine their own contextually and economically appropriate surveillance mechanisms, it may also lead to passive public health reporting systems that have typically been insensitive and unreliable for early detection of infectious disease outbreaks." (Sturtevant et al. 2007)
	Risks of implementing and running a public health surveillance system		
	Risk of inadequate legal regulation and governance structures for surveillance project	Inconsistent or overly complex legal guidance complicating effective and ethical implementation – especially for projects implemented across jurisdictions	"Although policies on data sharing exist in federal and other governmental agencies, a lack of standard language and processes related to data sharing across federal programs exists, with perhaps even less standardization at state and local levels. Efforts to standardize data sharing methods have been attempted throughout the U.S. Department of Health and Human Services but have not been realized in several instances. To date, guidance by the research and policy community on matters related to data policies and procedures at the national, state, and local levels has been inconsistent." (Bernstein & Sweeney 2012)<br><br>„Indeed, whilst in general there is a willingness to be in compliance with what can reasonably be expected from ethically correct conducted research, researchers are faced with a labyrinth of rules and guidelines, often open for interpretation, which leaves them worried about the fact that the legitimacy of the research which is ongoing might be challenged." (Dumez et al. 2008)
		No ethical review mechanism ensuring ethical obligations are followed– especially for projects involving online data sources	"Although the establishment of bodies responsible for the review of the ethics of surveillance need not mirror the already extant IRBs, as has been proposed by some experts on human subjects research, it is clear that some form of explicit, systematic, internal review is necessary." (Fairchild & Jones 2013)<br><br>"One approach calls for more systematic ethical review and oversight of public health surveillance that involves name-based reporting, even if it is practice rather than research." (Childress 2015)

		Ethics committees making inconsistent and delayed decisions (across jurisdictions)	"Inconsistent interpretation and application of privacy and ethical issues by IREBs delays and impedes research programs that could better inform us about chronic disease. […]The CPCSSN experience further highlights a need for Canada to develop a better process for research to obtain timely and consistent IREB approvals for multicentre research." (Kotecha et al. 2011)
	Risk of barriers hindering successful implementation or running of surveillance system	Lacking professionals adequately trained in health information technology	"The point can be made in the opposite direction: the failure to provide adequate training in health information technology will impede the evolution of HIT as a public health resource — a congenial way of saying that such a failure will prevent populations from realizing certain health benefits, that is, will allow people to be harmed." (Goodman 2010)
		Lack of security in areas of conflict	"Violence and resource limitations challenge the Afghan Ministry of Public Health's capacity to detect and investigate outbreaks. Lack of security is especially important in the volatile south, where 2 polio immunization workers were murdered and much of the region was inaccessible to immunization monitors in 2008, a worsening of conditions since 2007. Lack of security in the region also impeded efforts by the Ministry of Agriculture, Irrigation, and Livestock to control avian influenza H5N1 in poultry in 2007." (Chrétien et al. 2010)
		Lacking necessary infrastructural capacity (financial, technical, governance, human resources) - especially in developing countries	"Despite these incentives, surveillance activities vary across states, thereby leaving gaps in the surveillance networks. Such variations have been attributed to state and local differences in surveillance resources and infrastructure, in terms of financial, technological, and human capital."(Barnett & Sorenson 2011)<br><br>„The IHR requires national governments to implement and maintain outbreak surveillance systems at local or primary, state or intermediate, and national public health agency levels. This poses a formidable challenge to underdeveloped nations, which may not have adequate infrastructural capacity." (Sturtevant et al. 2007)
		Lacking political, societal or institutional commitment	"This inattention to the social situation of TB patients in general and migrants' living conditions in particular is in part due to a broader societal blindness regarding inequalities in health and the specific problems migrants seeking healthcare might face, an issue that was publicly debated in Germany only in

			the 2000s – contrary to France, where the issue of social rights for migrants has been much debated since the 1970s. This is even more true for undocumented migrants, who have been absent from much public discourse and official statistics in Germany for a long time, the health sector included, whereas French social movements early on put undocumented migrants' situation at centre stage in immigration debates." (Kehr 2012)
	Risk that burdens and benefits of surveillance systems are unfairly distributed	Developing countries disproportionately burdened by international surveillance effort	"Thus, even more than with the categorical initiatives of smallpox or polio eradication, there is considerable risk that public health services of resource-poor countries will feel the disruptive effects—more than the benefits—of joining the global surveillance agenda, through the very elusive nature of the threats that it covers." (Calain 2007)
	Further issues related to specific kinds of public health surveillance systems		
	Risks of surveillance systems relying on genetic profiles	Surveillance activity focusing too much on genes and not enough on other potential risk factors	"Many biologists, geneticists, medical researchers and social scientists alike argue that there has been an excessive and undeserved hype surrounding this genetic-arrow line of medical reasoning. Referred to variously as geneticization, genetic determinism, geno-mania or geneticism, this genetic focus identifies DNA as the exclusive component responsible for health and disease potential. Moreover, geneticism in the context of health care and disease not only distracts from the complexity of gene/environment and gene/gene interactions, but tends to completely ignore the socio-economic, cultural, and environmental conditions implicated in the aetiology of disease. In a context where the trajectory of health research and research funding is increasingly diverted toward to genetic determinants of disease and away from social and environmental factors, the hunt for the elusive gene thrives." (Poudrier 2002)
		Surveillance focusing on genetic profiles instead of other risk factors plays part in shifting (too much) responsibility to the individual	""Supplementing biomarkers of exposure by markers of genetic susceptibility tends to further frame the effects of exposure in terms of individual biology and, potentially, individual responsibility. It is the mobilisation into individual risk management that can be seen as part of an emerging "biological citizenship, as society, environmental policies and the health care system appear reconfigured according to bioscientific categories." (Bauer 2008)
	Risks of real-time surveillance systems	Surveillance system influences negatively the	"Issue a3b: Increased load on the local systems at the GP office, and correspondingly decreased responsiveness, caused by features in the

		usability of electronic medical records system other practitioners rely on	surveillance system. An example could be that too many requests and corresponding processes are executed simultaneously. For instance during outbreaks, many GPs would issue requests at the same time." (Henriksen et al. 2009)
	Conflicts in running vaccine safety surveillance systems during pandemics	Conflict of prioritizing early detection of adverse events or other effectiveness-related goals in distribution of vaccines	"Basic assumptions about pandemic influenza vaccine include high demand, limited supply, need for vaccine priorities, possible use of new technologies, and distribution prior to thorough evaluation of the product's safety profile. A vaccine safety surveillance system will have to anticipate possible problems while conducting effective surveillance of the product during its use. The need to detect adverse events in a timely manner will need to be balanced against issues of disease severity and vaccine efficacy." (Iskander 2005)
Issues in data collection, analysis and storage	Issues of protecting autonomy/the right to privacy		
	Risk of people not being adequately informed about usage of their data and drop-out options – especially where data from online sources is involved		"One concern about the system of priori notification is that patients may not see posters or leaflets in the clinic, or may not understand the significance of the information included. Opinions expressed by respondents in our current research suggest that information is not always displayed prominently and, in some cases, is not available at all. The plethora of posters adorning clinic walls may dissuade patients from reading them and could mean that they are unable to easily differentiate which posters are relevant to them. (Datta & Kessel 2009)<br><br>"Some sites that fall into this category are designed in part to provide tailored health feedback to users, but their business model is based on sharing the data they collect with commercial entities for research and other purposes. [...] It remains unclear whether consumers understand or are even aware that their supplied information can be used in research, whether for health-related surveillance, research, or commercial purposes." (Vayena et al. 2013)
	Risk of intentional breaches of privacy/confidentiality	Illegitimate authorities requesting data beyond what is ethically justifiable	"When consent cannot be obtained from the data subject, access should only be with the consent 'of a duly empowered legal authority acting with due process of the law' (p. 336) [20]. This requirement for legal sanction is, like 'consent', not enough on its own. In a regime that is corrupt, arbitrary, liable to prejudiced discrimination, totalitarian or otherwise acting beyond its moral authority; legal sanction may be given in circumstances where it should not, or

			denied when it should be given." (Fairweather & Rogerson 2001)
		Individuals involved in data processing releasing data without authorisation – especially where community members are involved in verbal autopsy	"There have been episodes in which public health data have been inadvertently released, such as an incident in Florida in which a health department official from Pinellas County reportedly showed a list containing 4000 names of people with HIV to patrons in a Tampa gay bar." (Fairchild & Jones 2013)<br><br>"Furthermore, it is not clear how confidentiality of the information obtained from VA is maintained, as the interviews are often conducted by a person living in the neighbourhood of the deceased." (Chandramohan 2005)
	Risk of unintentional breaches of privacy/confidentiality	Unauthorised access through inappropriate storage and transfer of data – especially where digital technology is used	"At the same time, it was acknowledged that the fallibility of health systems limited the extent to which uncertainties could be reduced. Concerns for safety and security have been exacerbated by a spate of recent incidents relating to individual data (which included civil servants' laptops containing patient data stolen from cars and trains), and this was reflected in comments in this context:. . . these systems betray you all the time to be honest. I know it can be violated and people break such things, like hacking into them and all that. (B) (female, age 27)." (Datta et al. 2013)<br><br>"In a concurring opinion, Justin Brennan affirmed that "most troubling" in this case was the issue of computer storage. While a new technology did not invalidate otherwise legitimate data collection and storage, he argued, "The central storage and easy accessibility of computerized data vastly increases the potential for abuse of that information, and I am not prepared to say that future development will not demonstrate the necessity of some curb on such technology." (Fairchild et al. 2007)<br><br>"Also for this threat it is difficult to anticipate likelihood. It relates to the possibility of wrong use of the system, and thus to usability aspects of the surveillance service's user interface: The system must be designed so that it is not too easy to place sensitive information into a message or to send a message to wrong address. For instance, if the Municipality Disease Prevention Doctor shall send (multicast) a message about a possible epidemiological outbreak to all GPs in his area, he must not, by accident, be able to also include

			another receiver." (Henrikson et al. 2009)
	Conflicts between obtaining informed consent (reflecting the values of confidentiality/ privacy/ respect for autonomy) and realizing public health benefit - especially in name- or personal-identifier-based reporting		"The ethical justification for public health surveillance without explicit patient consent presents itself as a challenge at the intersection of principles of clinical and public health ethics. The competing ethical priorities are the health care provider`s responsibility to protect patient confidentiality (derived from the ethical principle to respect the patient`s autonomy to have a say in the dissemination of her or his health information) and the public health authority`s responsibility to use the information to improve population health." (Lee 2012)<br><br>"The data generated by this important tool enable effective public health actions, but, insofar as the surveillance obtains personally identifiable information, there are concerns about threats to individual privacy and to the confidentiality of health-related information as well as about the lack of, or presence of only attenuated, individual consent." (Childress 2015)
	Risk of producing inadequate information to guide public health activities		
	Risk of collecting data that is not sufficiently accurate or complete	Collecting incorrect/fake data from user-supplied (online) data sources	“[Category f]: Reliability of user provided personal details - reliability of information derived from Twitter when some users use false or whimsical personal details to maintain anonymity.” (Conway 2014)
		Inadequate use of electronic collection system by professionals tainting data validity	"Another issue is accuracy of the original data. Health care workers ‘may be highly capable and competent, but if they lack the training necessary to use the program correctly, they may cause irreparable harm; [thus] the ideal we seek is that the user introduce clinically accurate data into the computer´. However, that ideal may well not be achieved, with the potential for serious detriment to the health and wider well being of the patient." (Fairweather & Rogerson 2001)
		Software errors or manipulations of electronic collection system reducing data validity	„Issue i1a, i1b: The surveillance system causes modification of data and relations in the local EHR system, resulting in wrong patient treatment. This could be caused by fake software modules doing this type of harm (see g2 above), or it could be caused by software errors in the surveillance system.” (Henriksen et al. 2009)
		Collecting unrepresentative data	"Reliable public health surveillance and research requires very broad public participation because any opting out introduces biases and distorts the picture

		only from parts of the population	that the data present. When affected groups are not included, a public health concern may be minimized, and when unaffected groups are not included, a public health concerns may be exaggerated. In other words, without broad public participation, selection bias occurs. Using data that is unrepresentative in any way can lead to public health policies that are poorly suited to the situation." (Rhodes 2006)
	Risks of health professionals not passing on data for analysis	Health professionals mistrusting legitimacy, usefulness and privacy of surveillance system	"Conversely it is vulnerable to changes in professional confidence and increasing work pressures on those who contribute. If doctors had serious doubts about the legitimacy of surveillance the easiest course of action would be for them to stop reporting, which would have harmful effects on the health of children and adults." (Verity & Nicoll 2002)<br><br>„Another particular problem arises when there are suspicions about the privacy of the patient record. 'Failure to record significant diagnoses and therapies . . . puts patients at risk.', yet because of the fear that patients may be harmed if records do not remain private, 'the practice of keeping 'double' records for patients [with psychiatric diagnoses] . . . has become widespread. Alternatively some clinicians . . . have created 'code language' to obscure the true content of clinical interactions' from those who were not present in the consulting room. While there may be legal protection of the privacy of the patient record in many jurisdictions, such protections are not sufficient if there are still suspicions on the part of either the patient or their doctor that records will not remain sufficiently private for a long time." (Fairweather & Rogerson 2001)
		Health professionals unwilling to carry administrative costs of surveillance system (without compensation)	"The sustained effort of public health officials to persuade physicians of their duties spoke to the ongoing problem of physician resistance, if not active opposition to reporting. […] Whereas Pierce pleaded ignorance, refusals to report more typically turned on the time and effort required for reporting, a burden that physicians viewed as a demand for unpaid labor and hence a deprivation of liberty and property." (Fairchild et al. 2007)
	Risk of inadequate analysis and interpretation of data	Gaps in evidence about subject hinder adequate interpretation	"With respect to the understanding and meaning of biomarkers, reviews often point to gaps in knowledge, data and validation that render interpretation difficult; for instance background and baseline values are not available for many ubiquitous environmental chemicals and their metabolites [5,31]."

			(Bauer 2008)
		Questionable reliability of methods used for data mining and meta-analysis	“Indeed, we now turn with increasing frequency to various forms of research synthesis to make sense of the data. The computational tools of meta-analysis and data mining will give us our best examples; they provide ways of eliciting conclusions, answers, or even mere suggestions from the apparent mess of data. They provide us with many case studies about whether and when to use a computer in making scientific decisions. Debates over meta-analysis, which often turn on its methods and reliability, remain important for any discussion of ethics in epidemiology in general, and ethics-computing-and-epidemiology in particular.” (Goodman & Meslin 2002)
		Meticulous analysis leads to harmful delays in times of emergency	"Processing data can result in delays for their release. Certain data collection programs do expend substantial resources on data cleaning and presentation and believe that data must be cleaned thoroughly and manipulated before they can be released and interpreted correctly by users. However, by the time data can be released, the value to public health surveillance programs might be limited if rapid response to a problem is necessary (e.g., to prevent spread of an infectious disease)." (Bernstein & Sweeney 2012)
	Risk of inadequately considering (vulnerable) subgroups in data collection		
	Risk of needs of (vulnerable) subgroups not becoming visible by inadequate data collection strategy	Surveillance based on online data sources excludes those without internet access	"Although there is widespread Internet access in the developed world, the digital divide remains a concern at the global level. Several countries report Internet usage percentages in single digits. Does health research based on such data have the inherent problem of bias and questionable generalizability? [...] It is also an ethical issue because particular groups are likely to be deprived of potential research benefits. Based on the general belief that health research using online information will translate into better health policy or medical advances, lack of online information could exacerbate existing health inequities." (Vayena et al 2012)
		Needs of (undocumented) migrants neglected	„Disease surveillance reports based on socially thin mandatory declarations remain silent about the distribution of TB, which is dissimilar among differently positioned migrant groups in society. The reports therefore struggle with important blind spots. As a consequence, those people most likely to be at risk for TB today – undocumented migrants and those without access to the healthcare system – remain invisible.“ (Kehr 2012)

		Needs of the poorest neglected	"In order for the data to cover all subgroups every home should be visited at least once a year and more frequently in the poorest areas." (Taylor 1992)
		Needs of people of colour neglected	"They also emphasize the importance of standard ethical requirements in research, including observation or surveillance as research. These include fairly selecting research participants to avoid ´unfair representation of the poor or people of color´." (Childress 2015)
	Risk of stigmatizing subgroups by data collection strategies that target only those subgroups	Strategies particularly targeting migrants	"Most social science studies examining the TB screening of migrants focus on their construction as a high-risk group, thus showing that targeted TB screening is not politically innocent but directly linked to national politics, and in particular to the politics of (border) control, These works contribute to the literature on the surveillance and control of at-risk groups through preventive public health measures. Analysing the discourses of immigration and the nation-state they describe the way public health policies construct different categories of risk, arguing that these categories justify enhanced interventionism and surveillance of migrants, revealing risk to be a central mechanism. It thus comes as no surprise that in these studies, the branding of migrants as being at high risk is found to have stigmatising and politically exclusionary effects." (Kehr 2011)
	Risks related to specific data collection strategies		
	Risks related to using verbal autopsy for data collection	Causing emotional distress in interviewees	"Verbal autopsy interviews, which rekindle the circumstances that led to the death of a close relative, can cause emotional distress. The degree of distress triggered by this process depends on various factors such as the age and relationship of the deceased, the circumstance of death, the culture of bereavement, time interval since death, and the counselling skills of the interviewer. For instance, interviewing a young woman who has lost her husband or child a few months earlier 'can end in tears'." (Chandramohan 2005)
		Data produced from interviews not reliable	"Fieldworkers came back with two often conflicting narratives from two sources for a particular death -– one from a relative/associate of the deceased and another unsolicited version of the terminal illness from a neighbour who volunteered the information to the fieldworker. Examples were cases of vague fevers reported by relatives but a history of HIV/AIDS related by neighbours, and history of vague somatic complaints reported by relatives but a history of

			suicide related by neighbours." (Mony & Vaz 2011)
	Risks related to using anonymous unlinked blood testing for surveillance	Foregoing the possibility to inform people about disease and treatment opportunities	"There are two main ethical issues associated with the programme and which have concerned commentators. […] The second is that, because of residual blood tested is unlinked from the individual who gave the sample, it is not possible to inform someone if their blood tests positive for HIV." (Datta & Kessel 2009)
Issues in data reporting, sharing and using for action	Issues of adequately protecting the right to privacy/confidentiality in data reporting and sharing		
	Risk of intentional breaches of privacy/confidentiality	Sharing data with commercial actors for their private benefit	"A potentially inappropriate use of the public database would therefore be for some sort of private gain or benefit. This is not a comment on or criticism of free enterprise or the free-market system. It is only to observe that public resources should not generally be used to benefit private interests." (Goodman & Meslin 2002)
	Risk of unintentional breaches of privacy/confidentiality	People publishing data are not adequately trained in data protection	"Occasionally, data stewards are reluctant to release data to others because they fear misuse of the data by those who are not well acquainted with its legal and technical limitations." (Bernstein & Sweeney 2912)
		Publicly disclosing data ensembles that allow indirect identification of individual	"Policies should restrict any portion of a release that might lead to indirect identification of individuals. For example, when releasing data about a community with few members of an ethnic group or age group in a specified geographic area, a person might be identified by the combination of these characteristics." (Heilig & Sweeney 2011)
		Publicly quoting social media streams	"[Category o:] Traceability of Twitter data - risk that tweets can be traced back to the original tweeter if reproduced verbatim in research work, threatening anonymity." (Conway 2014)
		Publicly releasing data that can be linked with other sources to identify individual	"We need to be mindful of the fact that disparate anonymized data can in some cases be concatenated or reanalyzed to elicit information about identifiable individuals." (Goodman 2010)
	Conflicts between protection of privacy/confidentiality and realizing public benefit in sharing data with actors outside the surveillance		"Opponents of such efforts feared the consequences of government collection of personal information, including discrimination and loss of privacy. Despite the formulation of the Model Public Health Privacy Act, key ethical questions regarding the uses of public health data remained unanswered. [...] May these data be shared within and among health departments and agencies? Finally,

	system		may they be shared with authorities or agencies outside of public health, for example, for the purposes of immigration, welfare benefits, or criminal law enforcement?" (Fairchild et al. 2007b)<br><br>"The goal is to have guidance on data release and sharing that balances the desire to disseminate data as broadly as possible with the need to maintain high standards and protect individuals' privacy and the confidentiality of the data." (Bernstein & Sweeney 2012)
	Issues of inflicting harm or restricting freedom when labelling individual/community as suffering from health issue		
	Risk of inflicting physical, social or emotional harm	Individuals experiencing psychological adverse effects	"It may be argued that the dead 'are no longer morally significant persons', and thus the only basis for respect towards the dead is the 'psychological harm to the living relatives'. While we are not convinced that this is the only basis, this basis alone could give rise to substantial obligations with respect to the treatment of the dead. People do like to think well of the dead, and could be anxious, for example, that some aspects of a patient record remained confidential, rather than be allowed to tarnish a reputation." (Fairweather & Rogerson 2001)<br><br>"The first ethical question - about the justifiability of collecting information without consent - can be distinguished from, though it is not unconnected with, questions about subsequent uses and risks of misuses of the collected information. The risks are mainly psychosocial in nature - embarrassment, stigmatization, difficulties in employment, and the like." (Childress 2015)
		Individuals/communities experiencing economic repercussion	"Nevertheless, this must be carefully considered against the potential imposition of harmful trade sanctions and adverse economic consequences that can result from earlier, broader, and more open information exchange, as occurred in Mexico after the swine flu outbreak in early 2009." (Barnett & Sorenson 2011)
		Individuals/communities experiencing stigmatization and discrimination	„To keep up with the spread of the virus, reporters evoked cultural accounts to explain the purportedly higher case fatality ratio in Mexico. Apparently, self-medication and delaying visits to appropriate health providers is a fault of ''Mexican'' national character. While Mexican health officials were often lauded by international health experts for being good global health citizens,

			reporters frequently cast Mexicans in general as unhygienic subjects and circulators of disease but not timely information. The circulation of such a perception possibly contributed to China's quarantining of Mexicans passing through its airports—or even living in ist cities. As Merrill Singer notes in his essay, such critiques and stereotypes of Mexicans morphed into attacks on Mexico and immigrants on right-wing U.S. talk radio shows." (Briggs & Nichter 2009)
		Physicians rejecting difficult patients to reduce problematic situations	"Another possible unintended negative outcome is that physicians will see to avoid difficult, noncompliant patients because of the implicit criticism in the notification." (Childress 2015)
		Individuals not accessing the care they need to protect their privacy	"In order to protect their privacy, individuals may avoid actions that benefit their health, such as HIV diagnosis or drug resistance testing. Not only could this behaviour be detrimental to the individual, but public health could be compromised due to the potential for an increase in new infections and increased transmission of resistant viruses." (Brooks & Sandstrom 2013)
	Conflicts between protection from psychosocial harm and realizing public health benefits	Protecting communities from stigmatization or benefiting them through additional resource	"It might not be possible, however, to eliminate all risk to vulnerable populations and the obligation to use the data to help these groups could outweigh limited or short-term harms. For example, the potential burden or stigma brought on by reporting aggregate data suggesting high rates of alcohol and drug use in a small community must be weighed against the potential benefit to this community through potential additional resources for drug and alcohol treatment services." (Heilig & Sweeney 2011)
	Risk of restricting freedom of choice	Individuals/communities facing coercive interventions or forms of punishment	"Tuberculosis and venereal disease were not, after all, just two more contagious diseases, but conditions to which tremendous moral opprobrium was attached. The consequences of disclosure could be devastating: […] Although rarely imposed, quarantine threatened the liberty of those with both VD and TB." (Fairchild et al. 2007)
	Conflicts between not limiting individual freedom and realizing public health benefit	Implementing coercive interventions that benefit the targeted individual	"In both instances, the specter of a malignant paternalism was raised. In the HIV debates, the specter of paternalism invited concerns about undue intrusions in the lives of those made vulnerable not only by disease but also by race and class and has threatened to obscure the fact that the new measures are far less about deciding for people and far more about providing for them.

			Indeed, it was the unmet need of marginalized populations that called forth the public health effort to fill a yawning gap." (Fairchild & Alkon 2007)
		Implementing coercive interventions that benefit other individuals	„More complex was the question of whether HIV/AIDS registries could be used to prevent infected health care workers from undertaking invasive procedures where there was a theoretical risk of blood-borne transmission. The question of whether such risks provided a justification for limits on practice informed the debate about the registry use." (Fairchild et al. 2007b)
	Issues of forgoing public health benefit by not adequately putting data to use		
	Risk of not using data (in time) for public health action	Lacking necessary resources to act upon data	"Unfortunately, these critics observe, the surveillance program generates personally identifiable information to be used for follow-up with patients, but it does not provide resources for their treatment or to bring others with uncontrolled diabetes into the healthcare system for diagnosis and treatment. As an article in the Lancet notes, in the absence of an infrastructure to provide comprehensive care for diabetes in the USA; this registry-based initiative can offer only a small step forwards for treatment of patients with diabetes." (Childress 2015)
		Other political interests given priority over public health goals	„How can it be that public health doctors and clinicians jointly agree not to treat a patient suffering from a treatable disease? The reasons are political rather than ethical, as the explanations of the public health doctor on the reasons for treatment failure show: she told me that at the inception of the programme there was a political consensus among immigration and public health administrations not to deport Roma patients in treatment and to maintain residential rights for those communities living in the same camp as the person diagnosed with TB. Yet the immigration authorities did not honour the agreement and so some treated patients or their family members were obliged to leave the French territory. Patients thus had to leave the country within a few weeks and were unable to complete treatment in France. In her explanation, the doctor clearly explained that interruption of treatment was not the Roma patients' fault. On the contrary, treatment interruption was seen as a logical effect of restrictive immigration policies in regard to Roma communities. Treatment completion was thus seen as a political impossibility and – in consequence – to begin treatment was to act irresponsibly in medical terms." (Kehr 2012)

	Risk of not sharing data with other actors	National protection hinders inter-governmental sharing of data	“In the case of SARS and avian flu, the Chinese and Indonesian governments, respectively, delayed or refused to share disease risk information with the rest of the world out of national protection.“ (Barnett & Sorenson 2011)
		Political interest in own visibility hinders sharing across institutions	"In other cases, data stewards are not willing to share data either for political or historical reasons or because they fear that if someone else has access to their data their program’s importance or visibility might be reduced." (Bernstein & Sweeney 2012)
		Insufficient resources invested in data sharing arrangement	"Resources are often used heavily in the front-end planning, data collection, and analytic phases of public health surveillance with proportionately less focus on data dissemination and translation phases. This could be related to insufficient resources that often make data sharing and investment in data sharing enhancements a lower priority than program work. Scarce resources also might make competition for funding contentious, which can result in lack of attention to relationship building at the highest levels that, if remedied, could facilitate future data-sharing arrangements." (Bernstein & Sweeney 2012)
		Incompatible processes for handling data hinder data sharing	"Data sharing can be impeded if coding, formatting, definitions, and methods differ substantially or if data are stored in incompatible formats. Resources are needed to manipulate, code, and transmit data to partners. Also, some analysis of data (e.g., analysis of trends) could be affected over time by changes in data collection, methods, and coding. These caveats often are not documented. Data sharing can be limited by the lack of user-friendly data dissemination tools or adequate and detailed documentation and distribution. If data descriptions are not available, well documented, and advertised, detailed data from federal data systems are much less likely to be used by others, including surveillance programs, to meet their specific data needs" (Bernstein & Sweeney 2012)
	Risk of not adequately communicating health risks to public	Unintentionally not providing all relevant information for action	"One is the issue of risk communication and the need to explain risks in a way that provides people with the information they need to determine appropriate action on the individual and community level." (Goldman et al. 1995)
		Deliberately communicating	"Apparently, those who made the decisions to withhold information and to promulgate false reports were more concerned with impressions, messages,

		misleading messages for political reasons	and promoting political ends than with promoting safety. In a similar way, false and misleading reports before, during, and after Hurricane Katrina cost lives and exaggerated the tragedy. These inaccurate and misleading communications undermined trust in government, in public health pronouncements, and in public health policy." (Rhodes 2006)
		Not finding the right level of alarm to induce adequate public reaction	"Later in the same article, the reporters restated this juxtaposition as a general principle of political conduct: ‘Finding the right mix of alarm and reassurance is a delicate task for an elected official.’ As the article continues, various ‘experts’ repeated that it is ‘dangerous’ for politicians to either ‘overreact’ or ‘underreact,’ but Obama ‘managed to get it just right.’" (Briggs & Nichter 2009)
