# Supplementary material for: Ethical issues in public health surveillance: a systematic qualitative review
Source: BMC Public Health. 2017 Apr 4;17:295. doi: 10.1186/s12889-017-4200-4 (PMC5381137; doi:10.1186/s12889-017-4200-4)
Supplement: Supplementary file 4 — Conditions that make foregoing informed consent procedures (more) acceptable with example quotes. (PDF 290kb) [file 12889_2017_4200_MOESM4_ESM.pdf]

Conditions that make foregoing informed consent procedures (more) acceptable with example quotes

**Justificatory conditions (Childress et al. 2002)**	
Code (Specification)	Example quote
**Effectiveness**	
Surveillance data is really put to use for public health purpose	“Second, the receipt of confidential medical reports must be used to serve public health ends. […] In short, the ethics, which justify limits on privacy and consent, require that these limits on individual rights will, in fact, serve the ends of public health.” (Bayer et al 2011)<br><br>"It is ethical to acquire surveillance data only if those data are put into use. The risks to privacy and liberty are justified only if the data are analyzed and disseminated for a public health purpose aimed at monitoring, preventing or controlling disease or improving or protecting health." (Heilig & Sweeney 2011)
**Necessity**	
Informed consent procedures reduce data validity by introducing bias	“For advocates of diabetes surveillance, however, complete ascertainment of cases was essential and a program that had to obtain consent would not be effective. Eran Bellin, Montefiore Medical Center’s director of outcomes analysis and decision support and son of the former New York City health commissioner who had written in favor of health department – based health-service quality improvement in 1977, was doubtless cognizant of the ways in which informed-consent requirements threatened to compromise the scientific validity of public health data. Although the program was not designed for 100 percent ascertainment of all cases in the city, being limited to labs that were equipped to report cases electronically, Bellin challenged those who pressed for an opt-in approach, arguing that any such protocol would result in a “grossly inaccurate undercount” and was “tantamount to undoing the entire effort”.” (Fairchild & Alkon 2007)<br><br>"Yet, informed consent has not been deemed necessary for the inclusion of an individual blood sample in the unlinked anonymous programme. There are several reasons for this. First, it has been argued that results may be biased if each individual whose blood is used is asked for explicit consent as it is possible, for example, that those more at risk of infection might be more likely than others to decline to take part, and this would adversely affect the epidemiological usefulness of the data." (Datta & Kessel 2009)
Less intrusive alternatives for collecting information not available	"Second, and relatedly, it must be that alternative methods that do not subordinate deep personal interests would not allow for sufficiently accurate assessment and similarly effective programs." (Rubel 2012)
Implementation of informed consent procedures not feasible	“Voluntariness and informed consent should not be required for population studies because the risks of public health surveillance, QA, and QI are typically negligible, opting out by any subjects introduces bias, and obtaining informed consent is often not feasible.” (Rhodes 2006)

Least infringement	
Opt-out option is provided instead	"Individuals whose residual blood is used in the national sero-surveillance programme for HIV, which has been operating in the UK since 1990, are not asked to give explicit consent for its inclusion. Literature in the form of posters and leaflets, produced by the Department of Health, explaining how the programme works and inviting patients to opt out if they are opposed to the use of their blood, is available for display in participating clinics. Those who have seen the literature are therefore able to opt out by telling the healthcare practitioner that they object to their blood being used in UAT, but practitioners are not expected to raise the subject during the consultation." (Datta et al. 2013)
Taking data against the will of the patient is preceded by attempt of convincing to give voluntarily (last resort)	"A patient should have effective control over his/her data and the ability to prevent any casual distribution that might be harmful to himself or herself, ensuring EPRs maintain nonmaleficence. There may need to be exceptions again, for example to combat contagion, but where the patient has at least an ordinary degree of rationality, strenuous efforts should be made at persuasion before release of the information is taken out of the individual's control." (Fairweather & Rogerson 2001)
Minimum amount of (preferably anonymised) data necessary for surveillance purpose is collected	"Additional requirements include whether the data elements collected without consent represent the minimal necessary interference. […] Current recommended practice is to collect the minimum number and simplest data elements necessary to meet the goals of the system to minimize risks to individuals, thus meeting the operating principles of imposing the least possible infringement." (Lee et al. 2012)<br><br>"In environments where the public is confident that government officials will use previously collected health information in a trustworthy manner, consent is not always required. […] They trust those authorities to acquire the evidence in the least intrusive ways possible. One of the ways to accomplish this is to render the data anonymous in salient respects. For instance, many public health surveillance efforts do not require the collection or storage of unique identifiers such as name, address, or Social Security Number; all that is needed is case information, context, and so forth." (Goodman & Meslin 2002)<br><br>"Neither the legal nor the ethical right to privacy is absolute. At most, they set a presumption against using personal identifiers in the collection and use of information; they establish a priority for the collection and use of anonymous or anonymized information whenever possible." (Childress 2015)
All relevant information about surveillance system is supplied to people affected	"However, they expressed unease about not being informed about the UAT process as they held that there should be a principle of transparency in dealings between government agencies and citizens. This did not necessarily mean that they thought consent should be sought from each individual – rather that people should be informed about the uses of their blood: There is something that I guess strikes me a little unethical about not informing every single person that gives blood, not just putting a poster up but actually telling them, by the way this is happening. (B) (female, age 27)" (Datta et al. 2013)<br><br>"Anyhow, also when individual informed consent would not be the key regulatory principle in some HBM research or surveillance practices, it is important that the person should be fully informed on what the research or practice is about, and on

	the reasons why no consent is asked for." (Dumez et al. 2008)
Data are maintained securely to minimize further risks	"At the surveillance centres we rely on the goodwill of health professionals for prompt reports of communicable diseases. We are aware that all data relating to individual patients must be secure. Some within the healthcare professions, and some patients, however, feel that patients' right to privacy overrides the need to maintain surveillance. In response to such concerns we are continuing to seek ways of reinforcing the security of data entrusted to us." (Evans & Ramsay 2001)
**Proportionality**	
Benefit to be realized/harm averted through surveillance activity considerable in probability and magnitude	"A provider must make a judgment about disclosure on the basis of the prima facie standing of autonomy with the probability and magnitude of harm. In the case of legally mandated case reporting, even without explicit patient consent, the probability and the magnitude of harm (resulting from not reporting) must be moderate to major on a population scale for reporting to be ethically justified. In the case of HIV reporting, the consequences of the absence of unbiased information about the incidence and distribution of cases could include numerous harms, including underappropriation of funds needed to treat and prevent infections, misallocated funds that are distributed to the most organized subgroup, and ultimately an increase in new infections and deaths." (Lee et al. 2012)<br><br>„"Where collection of health information does not redound to the benefit of the subject of the information, and where any health benefits to others are tenuous, small, or attainable in other ways, it would be difficult to justify the collection as fair to the persons whose conception of the good includes (or relies on) health information privacy." (Rubel 2012)"
Minimal Risks involved in data collection	"Because of the vanishingly small likelihood of risk, informed consent should not be required for these activities whenever general participation is needed and when obtaining agreement from individuals is not feasible." (Rhodes 2006)
Implementation of informed consent procedures would demand excessive resources	"Yet, informed consent has not been deemed necessary for the inclusion of an individual blood sample in the unlinked anonymous programme. There are several reasons for this. [...] Second, the need for clinicians to explain the programme to individual patients and to elicit signed consent would be time consuming and therefore expensive." (Datta & Kessel 2009)
No particularly sensitive information (e.g. mental or sexual health) is collected	"Realistically, however, patients will not be able to negotiate on an ad hoc basis with their health providers about the security and accessibility of their data. Instead, privacy solutions must take the form of standardised procedures built into the system itself that permit the partitioning of particularly sensitive categories of information (for example, sexual health, mental health, genetic counselling, sexual abuse and adolescent health data). In order to minimise the disincentives to seeking treatment, it would also be important for patients to be able to direct that certain clearly defined categories of data remained off-line, or to require their specific consent to be obtained before any health worker accessed that data category." (Magnusson 2002)
Potential public health benefits outweigh considerations of privacy protection	'It is acceptable to collect and use anonymous data assessing and predicting trends in infectious disease without consent as long as any invasion of privacy is reduced as far as possible.' However, even where non-anonymized data were collected for purposes of direct intervention, the Council asserted that 'The avoidance of significant harm to others who are at risk for a serious communicable disease may outweigh the consideration of personal privacy or confidentiality, and on this basis it can be ethically justified to collect non-anonymised data about individuals for purposes of implementing control measures.' (Bayer et

	al. 2011)<br><br>“Most emerging infectious diseases have been detected initially by an alert clinician who informs either the academics or the public health system. For many diseases, such “breaches of confidentiality” will be accepted by the majority of patients as they will be perceived as being in the interest of the safety and health of the wider population as a whole (including the family and friends of the “index case”).” (Ng & Tambyah 2011)
Implementing informed consent would set harmful standards for other surveillance programs	"The department compares diabetes to other conditions for which there is mandatory reporting and maintains that “requiring consent for reporting could set a hazardous precedent for other notifiable disease reporting, severely hindering the control of communicable disease outbreaks and the detection of environmental exposures.” (Rubel 2012)
**Public justification/engagement**	
The community/the public/those affected were engaged in decision.	“Thirdly, public consultation is needed to determine the ideal balance between, on the one hand, individual confidentiality and data protection and, on the other, the legitimate use of patient identifiable data without consent. Patients may not regard their contact with the National Health Service as constituting implied consent to the use of identifiable data about themselves for purposes other than their own medical care. However, there is a public interest in conducting observational research into diseases where little information is available and into audit of medical services which might be inadequate.” (Al-Shahi & Warlow 2000)<br><br>„Early engagement of partners and affected communities is recommended in the development of public health surveillance systems, especially when the data are sensitive or populations particularly vulnerable. It is often with the input from affected communities that decisions are made about what type of data should be collected in a surveillance system. Community engagement was used effectively during the 1990s to gain support for name-based HIV reporting, including in areas where initial opposition was vociferous, such as New York City and San Francisco, California. Operating principles of transparency, inclusiveness, and openness are addressed here.“ (Lee et al. 2012)
**Vulnerability (not in original list)**	
Data is collected to protect the health of children (who need special protection)	„In other cases, reporting may be justified by reasons that override the *pro tanto* reason not to restrict exercise of a claim to privacy. New York’s lead poisoning program, for example, requires the blood-lead levels of all children to be reported. As noted, one potential reason for restricting exercise of privacy claims is justified paternalism. Although determining when and whether paternalism is justified is a controversial endeavor, there is no question that it is much easier to justify it for children, and that could provide sufficient justification in the case of blood-lead reporting.“ (Rubel 2012)
No data from children is collected (because their privacy rights need special	"Third, special populations can elicit special protections. Thus, surveillance regimes involving children and reproduction have put a high premium on both confidentiality and informed consent." (Bayer & Fairchild 2000)

protection)	
**Legitimacy (not in original list)**	
Only legitimate entities trusted by the public collect surveillance data	"In environments where the public is confident that government officials will use previously collected health information in a trustworthy manner, consent is not always required. But that willingness is not to be presumed come what may: It is, we might surmise, a gift from citizens in open societies." (Goodman & Meslin 2002)
**Harm principles/unreasonable exercise requirement (not in original list)**	
Surveillance activity supposed to prevent harm to other individuals, not (only) same people being surveilled	"The first part of the argument is as follows: (1) Claims to privacy in health information with respect to state agencies are based on deep personal interests. (2) Subordinating a claim based on a deep personal interest is justified where the individual's exercise of that claim unreasonably threatens a basic interest of others within a community.(3) If exercising a claim based on a deep personal interest is likely to cause others serious injury, illness, or death, then exercising that claim unreasonably threatens a basic interest of others within a community. (4) Hence, where a person's exercising a claim to privacy in health information with respect to state agencies is likely to cause others serious injury, illness, or death, then it is justified to subordinate that claim. [...] (5) If exercising a claim based on a deep personal interest would not impose an unreasonable burden on others within a community, there is a pro tanto reason not to restrict exercise of that claim. (6) Thus, where exercising a claim to privacy in health information would not impose an unreasonable burden on others within a community, there is a pro tanto reason not to restrict exercise of that privacy claim." (Rubel 2012)<br><br>"An attorney described as representing health care groups concerned with medical privacy argued, ´This isn't smallpox. The state, or the city in this case, does not have a compelling interest in the health of an individual that overrides that individual's right to privacy´. Another individual likewise asked, ´What gives New York City the right to take my private information from me without my consent and usurp it as their own? Do I pose a bioterrorist threat? No. Is there some type of infectious disease threat? No. Is there an imminent threat that I will harm someone else? No´." (Fairchild & Alkon 2007)
